# Postnatal liver functional maturation requires Cnot complex-mediated decay of mRNAs encoding cell cycle and immature liver genes

**DOI:** 10.1242/dev.168146

**Published:** 2019-02-15

**Authors:** Toru Suzuki, Chisato Kikuguchi, Saori Nishijima, Takeshi Nagashima, Akinori Takahashi, Mariko Okada, Tadashi Yamamoto

**Affiliations:** 1 Laboratory for Immunogenetics, Center for Integrative Medical Sciences, RIKEN, 1-7-22, Suehiro-cho, Yokohama 230-0045, Japan; 2 Cell Signal Unit, Okinawa Institute of Science and Technology, 1919-1 Onna-son, Kunigami-gun, Okinawa 904-0495, Japan; 3 Division of Cell Proliferation, United Centers for Advanced Research and Translational Medicine, Tohoku University Graduate School of Medicine, 2-1 Seiryo-machi, Aoba-ku, Sendai, Miyagi 980-8575, Japan; 4 Laboratory for Integrated Cellular Systems, Center for Integrative Medical Sciences, RIKEN, 1-7-22, Suehiro-cho, Yokohama 230-0045, Japan; 5 Laboratory for Cell Systems, Institute for Protein Research, Osaka University, 3-2 Yamadaoka, Suita, Osaka 565-0871, Japan

**Keywords:** Liver development, Cnot complex, Deadenylation, mRNA decay, Mouse

## Abstract

Liver development involves dramatic gene expression changes mediated by transcriptional and post-transcriptional control. Here, we show that the Cnot deadenylase complex plays a crucial role in liver functional maturation. The *Cnot3* gene encodes an essential subunit of the Cnot complex. Mice lacking *Cnot3* in liver have reduced body and liver masses, and they display anemia and severe liver damage. Histological analyses indicate that *Cnot3*-deficient (*Cnot3^−/−^*) hepatocytes are irregular in size and morphology, resulting in formation of abnormal sinusoids. We observe hepatocyte death, increased abundance of mitotic and mononucleate hepatocytes, and inflammation. *Cnot3^−/−^* livers show increased expression of immune response-related, cell cycle-regulating and immature liver genes, while many genes relevant to liver functions, such as oxidation-reduction, lipid metabolism and mitochondrial function, decrease, indicating impaired liver functional maturation. Highly expressed mRNAs possess elongated poly(A) tails and are stabilized in *Cnot3^−/−^* livers, concomitant with an increase of the proteins they encode. In contrast, transcription of liver function-related mRNAs was lower in *Cnot3^−/−^* livers. We detect efficient suppression of Cnot3 protein postnatally, demonstrating the crucial contribution of mRNA decay to postnatal liver functional maturation.

## Abstract (precis)

**Summary:** Regulation of both mRNA transcription and stability plays a crucial role in postnatal liver development; in particular, Cnot complex-mediated mRNA decay is essential for postnatal liver functional maturation.

## INTRODUCTION

Liver development is regulated by various transcription factors (TFs), such as hepatocyte nuclear factor 4α (HNF4α), GATA and the Foxa family, which were initially identified as regulators of liver-specific genes. Studies of mice lacking these transcription factors demonstrated that these TFs are involved in cell-type specification or later organization of liver architecture during early developmental stages (Spear et al., 2006). Numerous changes in gene expression also occur from perinatal to adult stages, as hematopoiesis and proliferative activity are lost and genes involved in metabolism and detoxification are induced (Spear et al., 2006). Although much is known about involvement of TFs in both activation and repression during early liver development, regulation of mRNA levels, including mRNA decay in postnatal stages, has not been thoroughly addressed.

Mice lacking *Dicer1*, a gene encoding an enzyme for microRNA (miRNA) processing, and mice lacking *miRNA-122*, which is highly expressed in liver, show liver abnormalities, such as lipid accumulation, impaired metabolism and development of hepatocellular carcinomas (HCCs) (Sekine et al., 2009; Hsu et al., 2012; Tsai et al., 2012). Therefore, post-transcriptional activities of these molecules contribute to liver homeostasis and suppress tumor development in liver. Although these mice did not display overt defects in early liver development, *miR-122* regulates liver development in some contexts (Laudadio et al., 2012), underscoring the importance of mRNA decay in liver development.

A poly(A) sequence at the 3′end of mRNA influences mRNA stability and the frequency of translation. Shortening of poly(A) tails by deadenylation triggers mRNA decay from either the 5′ or 3′ end (Garneau et al., 2007). Cnot is the major cytoplasmic deadenylase complex that regulates mRNA turnover in eukaryotes from yeast to humans (Collart and Panasenko, 2012; Doidge et al., 2012). The 3′ untranslated region (3′UTR) of mRNAs has been implicated in regulation of mRNA decay. RNA-binding proteins that recognize specific sequences in the 3′UTR, such as AU-rich elements (AREs) or miRNA-binding sites, promote mRNA turnover (Lykke-Andersen and Wagner, 2005; Garneau et al., 2007; Filipowicz et al., 2008; Belloc and Méndez, 2008). The Cnot complex associates with the miRNA/Argonaute (Ago) complex or ARE-binding proteins, such as TTP and Zfp36L1, when recognizing target mRNAs (Zekri et al., 2009; Chekulaeva et al., 2011; Fabian et al., 2011, 2013; Huntzinger et al., 2013; Adachi et al., 2014; Takahashi et al., 2015).

In the mammalian Cnot complex, four catalytic subunits, Cnot6, Cnot6L, Cnot7 and Cnot8, have been identified as being crucial in regulating levels of target mRNA in various biological processes. Suppression of Cnot complex enzymatic subunits reduces cell growth in an activity-dependent manner (Morita et al., 2007; Aslam et al., 2009; Mittal et al., 2011). *Cnot7*-deficient mice display impaired spermatogenesis, enhanced bone formation and resistance to diet-induced obesity, although involvement of mRNA deadenylation has not been addressed in all these contexts (Berthet et al., 2004; Nakamura et al., 2004; Washio-Oikawa et al., 2007; Takahashi et al., 2015). A mutant Cnot8 protein that lacks most of the RNase domain affects dopaminergic neuron differentiation in zebrafish brain (Koch et al., 2014). Cnot1-3 and Cnot9-11 are non-catalytic subunits in the complex, but they seem to control deadenylase activity. In *Drosophila*, miRNA-dependent deadenylation is suppressed by Cnot1 depletion (Behm-Ansmant et al., 2006) and Cnot2 depletion affects the length of mRNA poly(A) tails (Temme et al., 2004). Knockdown of Cnot2 decreases deadenylase activity, leading to apoptosis in human cancer cell lines (Ito et al., 2011). Many studies specifically reveal crucial functions of Cnot3 in various biological processes. Cnot3 contributes to stemness or viability of embryonic stem cells and embryonic fibroblasts (Hu et al., 2009; Suzuki et al., 2015). Moreover, *Cnot3*-heterozygous (*Cnot3^+/−^*) or tissue-specific *Cnot3*-knockout mice show defects in heart function, energy metabolism, bone formation, B-cell development and adipocyte function (Neely et al., 2010; Morita et al., 2011; Watanabe et al., 2014; Inoue et al., 2015; Li et al., 2017; Yamaguchi et al., 2018). Because Cnot3 directly binds to the mRNA-binding protein Bic-C, Cnot3 is probably involved in recruitment of the Cnot complex to target mRNAs (Chicoine et al., 2007). Importantly, lack of Cnot3 is correlated with increased mRNA stability (Morita et al., 2011; Watanabe et al., 2014; Inoue et al., 2015; Suzuki et al., 2015; Yamaguchi et al., 2018). These intriguing findings provide a glimpse into the physiological importance of the Cnot complex. However, the roles of this complex in tissue development are poorly understood.

In this study, we genetically suppressed the Cnot complex in order to elucidate roles of mRNA decay in tissue development. We focused on liver development and generated mice that lack the *Cnot3* gene specifically in liver (Cnot3LKO mice). Cnot3LKO mice and their livers were smaller than normal, concomitant with abnormal liver structure and various pathologies. A number of mRNAs that were upregulated in *Cnot3^−/−^* livers had elongated poly(A) tails. Furthermore, they had longer half-lives in the absence of Cnot3. Genes encoding liver function-related molecules, such as metabolic enzymes, were expressed at very low levels due to insufficient transcription, indicating insufficient acquirement of adult liver characteristics. Therefore, we propose that Cnot complex-mediated mRNA decay is essential for postnatal liver functional maturation.

## RESULTS

### Albumin promoter-driven Cre recombinase efficiently suppresses Cnot3 in postnatal liver and induces differences in histology and gene expression

**Fig. 1.**
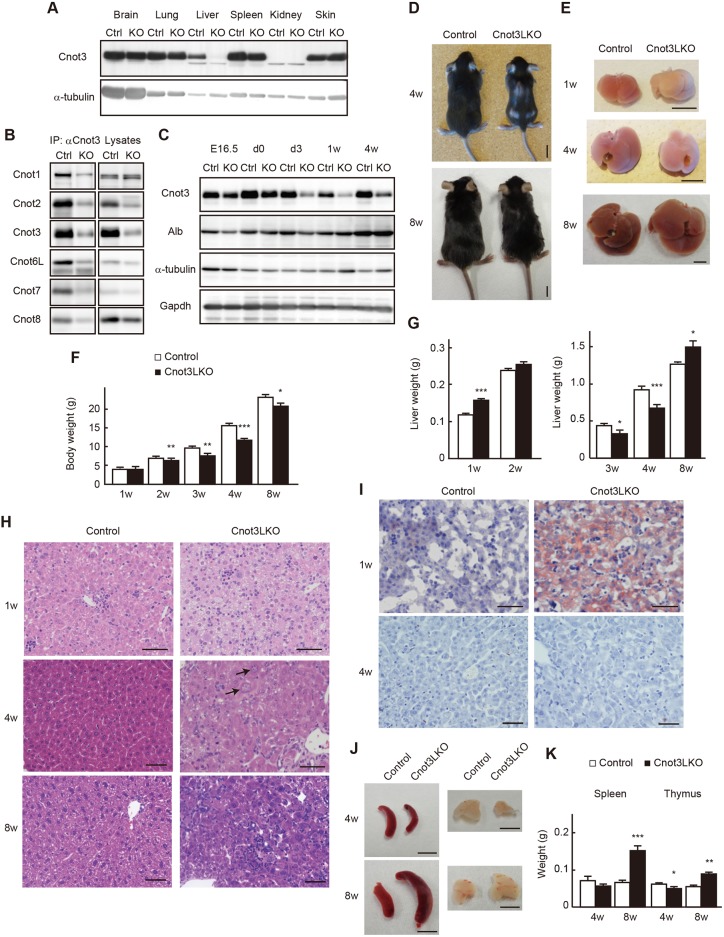
**Liver-specific Cnot3 suppression results in decreased body mass and abnormal liver histology.** (A) Immunoblot of tissue lysates from 4-week-old (4 w) control (Ctrl) and Cnot3LKO (KO) mice. (B) Immunoblot of anti-Cnot3 immunoprecipitates and lysates from 4 w Ctrl and KO livers. (C) Immunoblot of liver lysates from Ctrl and KO mice. (D,E) Appearances of whole body (D) and liver (E) of control and Cnot3LKO mice. (F,G) Body (F) and liver (G) weights of control and Cnot3LKO male mice (1-4 w, *n*=7; 8 w, *n*=5). (H) Hematoxylin and Eosin-stained livers from control and Cnot3LKO mice. (I) Oil Red-O stained livers from control and Cnot3LKO mice. (J) Appearances of spleen (left panels) and thymus (right panels) from control and Cnot3LKO mice. (K) Spleen and thymus weights of control and Cnot3LKO male mice (4 w, *n*=7; 8 w, *n*=5). Scale bars: 10 mm in D; 5 mm in E,J; 50 μm in H,I. Data are mean±s.e.m. *P<0.05, **P<0.01, ***P<0.001.

Although *Cnot3*-null mice do not develop past embryonic day 6.5 (E6.5), *Cnot3^+/−^* mice develop to adulthood and are lean, due at least in part to enhanced energy metabolism in liver (Morita et al., 2011). To identify physiological roles of Cnot3 in liver development and function, we crossed albumin promoter-driven Cre recombinase (Alb-Cre) transgenic mice with mice carrying the floxed allele of *Cnot3* to obtain Cnot3LKO mice. Immunoblot analyses demonstrated liver-specific suppression of Cnot3 (Fig. 1A). Consistent with results in Cnot3-depleted MEFs or B-cells (Inoue et al., 2015; Suzuki et al., 2015), levels of most other subunits also decreased upon Cnot3 suppression (Fig. 1B). Consequently, intact Cnot complex was largely reduced in Cnot3LKO mouse livers (Fig. 1B). We used an mTmG reporter transgene (Muzumdar et al., 2007) to monitor when and where Alb-Cre-mediated recombination is induced. In mice containing the transgene, recombination-induced cells express green fluorescent protein (GFP) at the membranes, whereas the others express tdTomato at the membranes. We generated (+/+):Alb-Cre and Cnot3LKO mice possessing the transgene and examined expression of the reporter proteins. In both control and Cnot3LKO mice, many cells expressed GFP in livers of E16.5 and newborn (d0) mice, although we detected a significant number of tdTomato-expressing cells that included hematopoietic cells (Fig. S1). In E12-16 mouse livers, bipotential hepatoblasts are the major Alb-expressing cells, which also express α-fetoprotein (Afp), delta-like 1 homolog (Dlk1) and a cholangiocyte marker: cytokeratin 19 (CK19) (Tanaka et al., 2009; Gordillo et al., 2015). They correspond to GFP-expressing cells in livers from mice possessing an mTmG reporter transgene. They multiply and start to differentiate into hepatocytes or cholangiocytes during these stages (Tanaka et al., 2009; Gordillo et al., 2015). From 3 days to 4 weeks after birth, most cells in the livers expressed GFP in both control and Cnot3LKO mice. Cells expressing tdTomato would be non-hepatic cells, such as cholangiocytes. These data suggest that Alb-Cre-mediated recombination is induced, but not completely, at E16.5 and sufficiently induced at postnatal day 3 (d3) and later. Immunoblots of liver lysates reflected the results, i.e. Cnot3 decreased by about half at E16.5 and was largely downregulated at d3 and later (Fig. 1C). We observed similar histological patterns in E16.5 livers and slight differences as indicated by the appearance of cytoplasmic vacuoles in d3 *Cnot3^−/−^* livers (Fig. S2A). Then, we compared gene expression profiles using microarray analysis of total RNAs from E16.5 and d3 livers. Consistent with histological analysis, control and *Cnot3^−/−^* livers showed almost identical gene expression profiles at E16.5 (Fig. S2B). In contrast, we detected several differences in gene expression profiles at d3, ∼1400 mRNAs were upregulated more than 1.5-fold and 700 were downregulated more than 1.5-fold in *Cnot3^−/−^* livers, respectively (Fig. S2B). Therefore, analysis of Cnot3LKO mice enables us to evaluate roles of Cnot3 and mRNA deadenylation mainly after birth rather than during embryonic liver development.

### Liver-specific Cnot3 suppression induces decreased body and liver weight, inflammation, and temporal lipid accumulation in liver

**Fig. 2.**
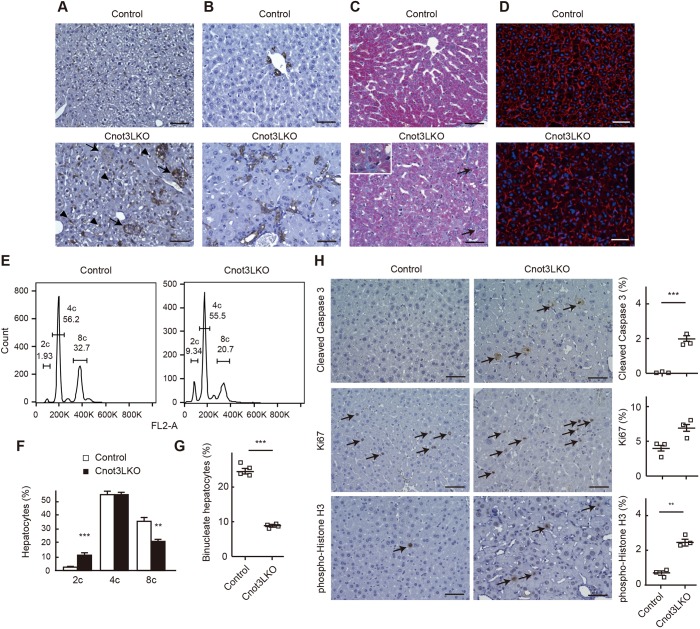
**Increased bile duct reaction, apoptotic cells, dividing cells and mononucleate cells are evident in *Cnot3*^−/−^ livers.** (A-D) Immunohistochemistry for F4/80 (A) and CK19 (B), Masson's trichrome staining (C) and actin staining (D) of livers from 4-week-old (4 w) control and Cnot3LKO mice. Arrowheads and arrows in A indicate macrophages and those engulfing hepatocytes, respectively. Arrows in C indicate weak fibrotic regions. Magnified views of representative areas are shown in the inset in C. (E,F) Identification of cells with diploid (2c), tetraploid (4c) and octaploid cellular content (8c), corresponding to diploid, tetraploid and octaploid hepatocytes by FACS analysis of hepatocytes stained with PI. Representative profiles are shown in E. (F) Graph shows the percentage of each population quantified using FlowJo (*n*=5, 4 w). (G) The percentage of binucleate hepatocytes in 4 w control and *Cnot3*^−/−^ livers (*n*=4, more than 350 cells were counted). (H) Immunohistochemistry for cleaved caspase 3 (top), Ki67 (middle) or pHH3 (bottom) in 4 w control and *Cnot3*^−/−^ livers. Arrows indicate stained cells. Graphs show percentages of hepatocytes positive for each immunostaining. Five different fields (total of around 1000 cells) in each section were counted (*n*=3). Data are mean±s.e.m. Scale bars: 50 μm. **P<0.01, ***P<0.001.

Cnot3LKO mice generally survived, and both Cnot3LKO males and females were fertile. Mice born from matings between Cnot3LKO males and Cnot3LKO females also reached adulthood. A small percentage (less than 5%) of Cnot3LKO mice were extremely small and sometimes died, probably due to severe liver damage. These extremely small mice were not included in subsequent analyses. Cnot3LKO mice were significantly smaller than their control littermates (Fig. 1D,F). Control and Cnot3LKO mice had similar weights at 1 week of age, but a significant weight difference was evident by about 2 weeks. That difference continued to increase as mice grew up to 8 weeks (Fig. 1F). At 1 week of age, livers of Cnot3LKO mice were significantly larger than those of control mice (Fig. 1E,G), although the amount of increased liver mass was not enough to affect total body weight. Livers of 1-week-old (1 w) Cnot3LKO mice were noticeably white, and histological analysis showed that *Cnot3^−/−^* hepatocytes had cytoplasmic vacuoles, suggestive of lipid accumulation (Fig. 1E,H, top panels). Oil Red-O staining confirmed the presence of numerous lipid droplets, which explained the increased weight of *Cnot3^−/−^* livers (Fig. 1I, upper panels; Fig. S3A). Lipid accumulation decreased sharply at 2 weeks of age (Fig. S3). When Cnot3LKO mice reached ∼3 weeks of age, their livers were smaller than those of control mice (Fig. 1E,G). Although *Cnot3^−/−^* livers displayed a pale color at that age, lipid accumulation was scarcely detectable (Fig. 1I, lower panels); hence, the pale color was mainly due to other reasons (described below). Thereafter, other abnormalities appeared. In livers of control mice at 4 and 8 weeks of age, hepatocytes displayed uniform size and morphology, whereas in those of Cnot3LKO mice at the same ages, sinusoids were compressed by hepatocytes of various sizes and irregular morphology (Fig. 1H, middle and bottom panels). Furthermore, we observed various pathological abnormalities in *Cnot3^−/−^* livers at both 4 and 8 weeks of age, such as necrosis, inflammation, macrophage infiltration, bile duct proliferation and extramedullary hematopoiesis (Fig. S4A). Infiltration of macrophages into hepatocyte areas was confirmed using immunohistochemistry (IHC) for F4/80 and CD45 (Fig. 2A and Fig. S4B). We found that 8 w Cnot3LKO mice had significantly enlarged livers, thymus glands and spleens, probably caused by enhanced and continued inflammation in the livers (Fig. 1E,G,J,K). Other tissues appeared normal.

### More numerous biliary ductular cells, mononucleate cells, apoptotic cells and dividing cells in *Cnot3*^−/−^ livers

In normal mice, in response to liver injury, biliary epithelial cells and oval cells, both of which express CK19, start to proliferate (Alvaro et al., 2007; Tanaka et al., 2011). That cellular response is correlated with periportal fibrosis (Alvaro et al., 2007). Necrotic death of hepatocytes and bile duct proliferation in *Cnot3^−/−^* livers (Fig. S4) led us to examine changes in nonparenchymal cells. *Cnot3^−/−^* livers exhibited a prominent increase of CK19-positive cells (Fig. 2B and Fig. S5) and displayed very mild fibrosis (Fig. 2C). Actin staining in hepatocytes revealed cytoskeletal disorganization in *Cnot3^−/−^* livers, possibly relevant to irregular hepatocyte morphology and alignment (Fig. 2D). We found the different distribution of mononucleate and binucleate hepatocytes between genotypes, and then analyzed propidium iodide (PI)-stained hepatocytes using flow cytometry. This analysis allowed us to identify and quantify diploid, tetraploid and octaploid hepatocytes. A small percentage of control hepatocytes were diploid, whereas the majority were tetraploid and octaploid (Fig. 2E,F). In contrast, the ploidy spectrum of *Cnot3^−/−^* hepatocytes was clearly different. Many more *Cnot3^−/−^* hepatocytes were diploid and the percentage of *Cnot3^−/−^* octaploid hepatocytes was significantly less than that of controls (Fig. 2F). In liver, diploid hepatocytes are mononucleate, and tetra- and octaploid hepatocytes are mononucleate or binucleate. We observed that *Cnot3^−/−^* livers had a smaller percentage of binucleate hepatocytes than controls (Fig. 2G), which is correlated with an increase of diploid hepatocytes. We also detected apoptotic death of hepatocytes by IHC for cleaved caspase 3 in *Cnot3^−/−^* livers (Fig. 2H, top), indicating that *Cnot3*^−/−^ hepatocytes undergo several forms of cell death. Immunohistochemistry for a proliferation marker, Ki67 antigen, showed that both control and *Cnot3^−/−^* hepatocytes had similar proliferative activity at this developmental stage (Fig. 2H, middle). In contrast, the number of mitotic cells increased in *Cnot3^−/−^* livers, as indicated by phospho-histone H3 (pHH3)-positive cells (Fig. 2H, bottom). We confirmed that the cleaved caspase 3, Ki67 and pHH3-positive cells expressed Alb using immunofluorescence (Fig. S6).

### Cnot3LKO mice display anemia and severe liver damage

**Fig. 3.**
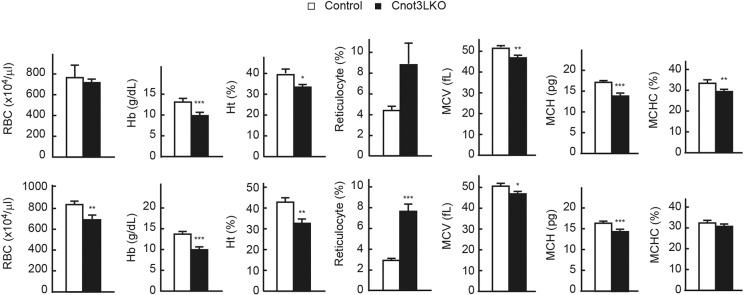
**Cnot3LKO mice display iron-deficiency anemia.** Concentrations of red blood cells (RBCs), hemoglobin (Hb), hematocrit (Ht) and reticulocytes were measured in blood from 4-week-old (4 w) (upper graphs), and 7 w and 8 w (lower graphs) control and Cnot3LKO mice (control: *n*=6 at 4 w, *n*=4 at 7 w, *n*=3 at 8 w; Cnot3LKO: *n*=6 at 4 w, *n*=3 at 7 w, *n*=4 at 8 w). MCV, MCH and mean corpuscular hemoglobin concentration (MCHC) were calculated from RBC, Hb and Ht values. Data are mean±s.e.m. *P<0.05, **P<0.01, ***P<0.001.

The pale color of *Cnot3^−/−^* livers in the absence of obvious lipid accumulation (Fig. 1E) led us to examine whether there were changes in blood components. Blood tests revealed that, compared with control mice, hemoglobin levels and hematocrit decreased in 4-, 7- and 8-week-old Cnot3LKO mice, and the number of red blood cells, hemoglobin levels and hematocrit decreased, while the percentage of reticulocytes increased in 7- and 8-week-old Cnot3LKO mice (Fig. 3). Both mean corpuscular volume (MCV) and mean corpuscular hemoglobin (MCH) were lower, but we detected no obvious hemorrhage in Cnot3LKO mice. Therefore, Cnot3LKO mice suffered from anemia, likely due to iron deficiency. Biochemical analysis of blood showed that iron levels were significantly reduced in Cnot3LKO mice (Table 1). Moreover, alanine transaminase (ALT), aspartate aminotransferase (AST) and alkaline phosphatase (ALP) levels were strongly elevated in Cnot3LKO mice. Therefore, loss of Cnot3 resulted in abnormal liver functional maturation concomitant with cell death, anemia and characteristics of damaged liver.

**Table 1.**
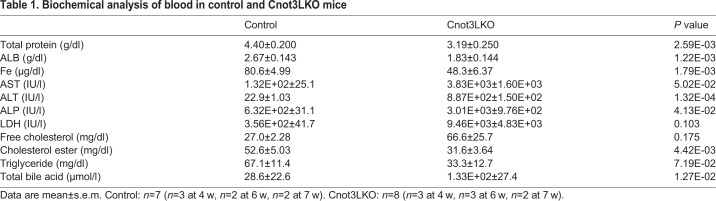
**Biochemical analysis of blood in control and Cnot3LKO mice**

### Gene expression differences in *Cnot3*^−/−^ livers show enhanced inflammation, cell-cycle progression and immature liver function

**Fig. 4.**
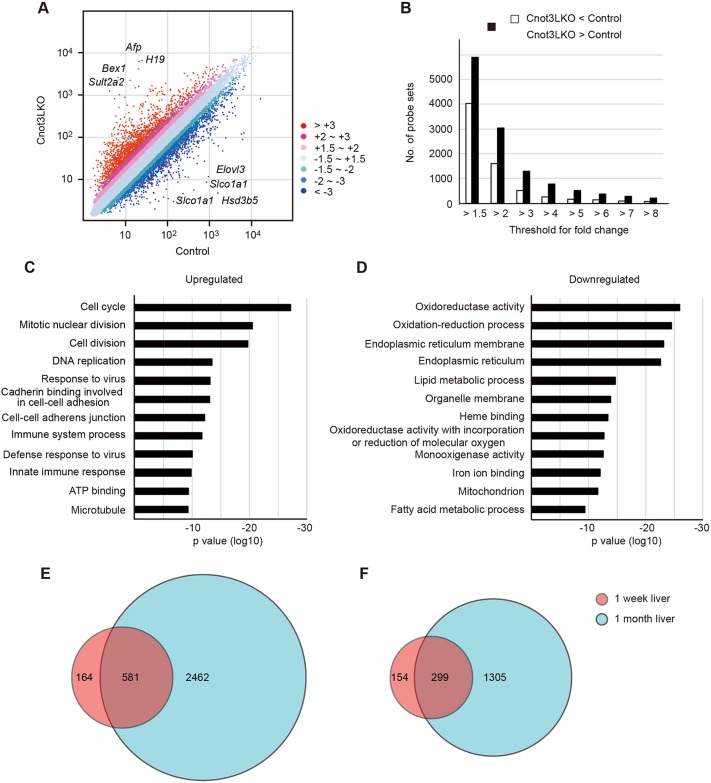
**Gene expression levels and GO analysis show upregulation of immunoresponses and cell-cycle events, and downregulation of liver function in *Cnot3*^−/−^ livers.** (A) Scatter plot of mRNA expression in livers from 4-week-old (4 w) control and Cnot3LKO mice (*n*=2). Eight transcripts showing the largest differences are indicated. (B) Bar plot of the number of probe sets upregulated (black bars) or downregulated (white bars) with changes in *Cnot3*^−/−^ livers relative to controls. (C,D) GO analysis of mRNAs upregulated >2-fold (C) or downregulated (D) >2-fold in *Cnot3*^−/−^ livers compared with controls. Bar charts of the top 12 GO terms ranked by *P* value are shown. Gene lists included in each GO term are summarized in Table S2. (E,F) Venn diagram illustrating the overlap of genes upregulated >2-fold (E) or downregulated (F) >2.0-fold in *Cnot3*^−/−^ livers between 1 w (circled red) and 4 w (circled blue).

To define gene expression changes and to investigate their relationships to abnormalities observed in *Cnot3^−/−^* livers, we performed a microarray analysis. We prepared total RNA from livers of control and Cnot3LKO mice. At 1 week of age, levels of 2% of the probes were upregulated more than 2-fold and levels of 1.2% of the probes were downregulated more than 2-fold in *Cnot3^−/−^* livers compared with controls (Fig. S7A). At 4 weeks of age, the situation was further exacerbated, with 7.7% of probes upregulated more than 2-fold and 4.1% downregulated more than 2-fold (Fig. 4A). The number of probes upregulated is nearly twice the number of those downregulated at both ages (Fig. 4B and Fig. S7B). This increase in upregulated genes is probably caused by loss of Cnot3 function (mRNA deadenylation), as previously described (Chicoine et al., 2007; Morita et al., 2011; Watanabe et al., 2014; Suzuki et al., 2015; Inoue et al., 2015). To characterize differences in gene expression at different developmental stages of *Cnot3^−/−^* livers, we performed gene ontology (GO) analysis using lists of upregulated and downregulated genes. Functional annotation of upregulated genes at 1 week of age showed enrichment of genes associated with immunoresponses and cell-cell contact (Fig. S7C and Table S1). Those GO terms were also enriched at 4 weeks of age and many genes corresponding to these terms overlapped between 1 and 4 weeks (Fig. 4C,E). In contrast, genes associated with cell cycle, cell division and DNA replication were enriched in *Cnot3^−/−^* livers, especially at 4 weeks of age (Fig. 4C). That explains the greater difference of upregulated genes from 1 to 4 weeks of age (Fig. 4E and Fig. S7E). In particular, gene lists for the GO term ‘cell division’ included a number of mRNAs encoding molecules that promote cytokinesis and chromosome segregation (Table S2), which may lead to increased number mononucleate diploid hepatocytes in *Cnot3^−/−^* livers (Fig. 2). On the other hand, functional annotation of downregulated genes showed enrichment of genes encoding liver function-related molecules, such as oxidoreduction, endoplasmic reticulum function and lipid metabolism at both ages (Fig. 4D and Fig. S7D). The number of downregulated genes also increased from 1 to 4 weeks of age (Fig. 4F), and most of those were liver function-related genes (Fig. S7F). These data suggest that expression of genes, induced in normal mouse liver after 1 week of age is dysregulated in the absence of Cnot3, and, consequently, *Cnot3^−/−^* livers do not acquire mature liver characteristics, even after weaning.

### Upregulation of immunoresponse-related genes and cell cycle-regulating genes, and downregulation of various metabolic genes in *Cnot3*^−/−^ livers

**Fig. 5.**
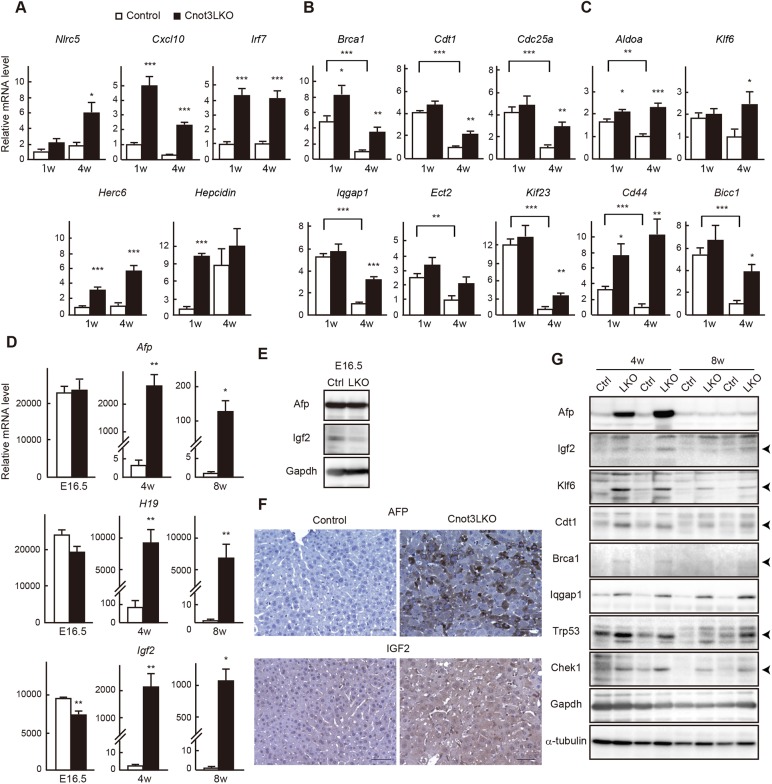
**Genes involved in immunoresponses, cell cycle and early tissue development manifest increased expression in postnatal *Cnot3*^−/−^ livers.** (A-D) qPCR analysis of mRNAs in livers from E16.5, 1-week-old (1 w) and 4 w control and Cnot3LKO mice. *Gapdh* mRNA levels were used for normalization. Expression levels of genes in control mice (1 w in A, 4 w in B,C; 8 w in D) are set to 1 (*n*=5). Data are mean±s.e.m. *P<0.05, **P<0.01, ***P<0.001. (E,G) Immunoblot of tissue lysates from control (Ctrl) and Cnot3LKO (KO) mice. (F) Immunohistochemistry for Afp and Igf2 using 4 w control and *Cnot3*^−/−^ livers. Scale bars: 50 μm.

We then validated microarray results using quantitative PCR (qPCR) analysis and confirmed that immunoresponse-related transcripts (*Nlrc5*, *Cxcl10*, *Irf7* and *Herc6*) were more abundant in *Cnot3^−/−^* livers at 1 or 4 weeks of age (Fig. 5A). The hepatic peptide hormone hepcidin is a key regulator of iron homeostasis and its overproduction is relevant to anemia associated with inflammation (Ganz and Nemeth, 2012). We detected a significant elevation of *hepcidin* mRNA expression in *Cnot3^−/−^* livers at 1 week of age (Fig. 5A), suggesting that the increase was, at least in part, responsible for an iron-deficiency anemia of Cnot3LKO mice (Fig. 3). Consistent with microarray results, cell cycle-related genes (*Brca1*, *Cdt1*, *Cdc25a*, *Cdc25b*, *Iqgap1*, *Ect2*, *Kif23*, *Chek1* and *Trp53*) were expressed more highly in *Cnot3^−/−^* livers compared with controls, mainly at 4 weeks of age (Fig. 5B and Fig. S8A). Iqgap1 and the centralspindlin complex proteins (Ect2 and Kif23) promote cytokinesis and have been identified as molecules that reduce binucleate hepatocytes in liver lacking *miR-122* (Hsu et al., 2016), suggesting that they contribute to increase of mononucleate hepatocytes in *Cnot3^−/−^* livers. We found that levels of around 7000 mRNAs decreased more than 3-fold from 1 to 4 weeks in postnatal control livers using our microarray results. Comparison of mRNAs showing maturation-dependent decrease with upregulated mRNAs in *Cnot3^−/−^* livers (>2-fold increase compared to controls in Fig. 4A) displayed substantial overlap. Note that more than 65% of upregulated mRNAs in *Cnot3^−/−^* livers corresponded to mRNAs that decrease from 1 to 4 weeks in control livers (Fig. S9A). mRNAs that show both upregulation in *Cnot3^−/−^* livers and a maturation-dependent decrease included a number of growth-promoting genes and cell cycle checkpoint genes. The top three GO terms were cell cycle, mitotic nuclear division and cell division (Fig. S9B and Table S3). Consistent with this, the results of qPCR showed that levels of cell cycle-related mRNAs decreased less efficiently from 1 to 4 weeks in *Cnot3^−/−^* livers than in control livers (Fig. 5B and Fig. S8A). These data suggest that postnatal liver functional maturation involves a decrease of cell cycle-related mRNAs, and Cnot3 suppression results in insufficient reduction of those mRNAs. Microarray results also revealed that *Klf6*, *Cd44*, *Bicc1* and *Aldoa* mRNAs as well as fetal liver genes (*Afp, H19* and *Igf2*) are upregulated in *Cnot3^−/−^* livers. Klf6 is responsible for hepatocyte specification (Zhao et al., 2010). In the fetus, the liver is the primary organ of hematopoiesis, and Cd44, a surface marker of hematopoietic stem cells, indicates the state of hematopoiesis in fetal liver (Ohata et al., 2009). The Cnot complex regulates *Bicc1* mRNA in *Drosophila* development (Chicoine et al., 2007), although its role in mammalian liver development has not been addressed. These transcripts were expressed significantly more in *Cnot3^−/−^* livers than in controls (Fig. 5C). *Afp*, *Igf2* and *H19*, which are well-known oncofetal genes, are expressed at high levels in immature liver and decrease as the liver develops. Expression of *Afp* mRNAs and *H19* was comparable in embryonic liver between control and Cnot3LKO mice, and *Igf2* mRNA expression was slightly lower in *Cnot3^−/−^* livers (Fig. 5D). Immunoblots of Afp and Igf2 showed identical results (Fig. 5E). Although these proteins were downregulated compared with the embryonic period in both genotypes, they were expressed at significantly higher levels in 4-week-old *Cnot3^−/−^* livers than in controls (Fig. 5D). Indeed, immunohistochemistry for Afp and Igf2 revealed that most *Cnot3^−/−^* hepatocytes expressed them at detectable levels (Fig. 5F). We confirmed that upregulation of cell cycle-related and fetal liver mRNAs resulted in increased protein levels (Fig. 5G). Furthermore, upregulation of those molecules was observed in 8-week-old mice (Fig. 5D,G and Fig. S8B).

**Fig. 6.**
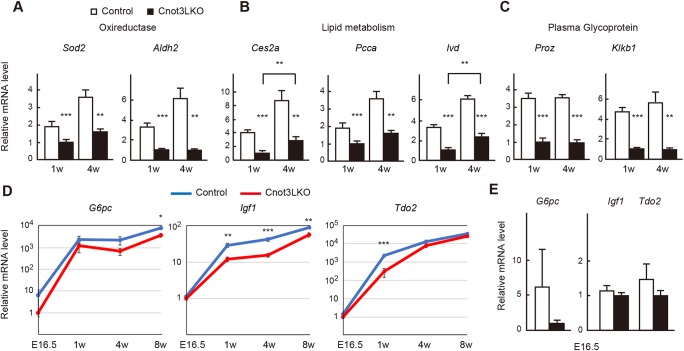
**Genes involved in liver function display decreased expression in postnatal *Cnot3*^−/−^ livers.** (A-D) qPCR analysis of mRNAs in livers from E16.5, 1-week-old (1 w) and 4 w control and Cnot3LKO mice. *Gapdh* mRNA levels were used for normalization. Expression levels of genes in 1 w (A-C) and E16.5 (D) Cnot3LKO mice are set to 1 (*n*=5). (E) Relative mRNA levels at E16.5 in D are shown as bar charts. Data are mean±s.e.m.. *P<0.05, **P<0.01, ***P<0.001.

Next, we performed qPCR analysis of genes downregulated in *Cnot3^−/−^* livers and found that mRNAs encoding oxidoreductases (Sod2 and Aldh2), lipid metabolism enzymes (Ces2a, Pcca and Ivd) and liver-synthesized plasma glycoproteins (Proz and Klkb1) were expressed significantly less in *Cnot3^−/−^* livers (Fig. 6A-C). Nevertheless, levels of some lipid metabolic enzymes increased significantly from 1 to 4 weeks, even in the absence of Cnot3 (Fig. 6B). Again, these transcripts showed similar expression differences in 8-week-old mice (Fig. S8C). Furthermore, we detected decreased expression of *G6pc*, *Igf1* and *Tdo2* mRNAs between control and *Cnot3^−/−^* livers at several developmental stages, although they obviously increased in both genotypes as mice matured (Fig. 6D). Therefore, transcriptional programs are dysregulated or delayed in *Cnot3^−/−^* livers.

### *In vitro* differentiated *Cnot3^−/−^* fetal hepatocytes show different gene expression profiles and stabilization of cell cycle-related mRNAs

Levels of *G6pc*, *Igf1* or *Tdo2* mRNAs, as well as fetal liver genes and proteins, were comparable at E16.5 between control and Cnot3LKO mice (Figs 5D,E and 6E). These appeared to be due to incomplete suppression of Cnot3 at E16.5 (Fig. 1C). We prepared primary fetal hepatocytes from both genotypes and compared their differentiation properties. Control and *Cnot3^−/−^* fetal hepatocytes were viable and similarly differentiated, as shown by increases in levels of *G6pc* and *Tdo2* mRNAs and Alb protein (Fig. S10A,B). Unexpectedly, fetal liver mRNA levels did not decrease during *in vitro* differentiation (Fig. S10A). Consistent with this, Afp protein levels did not decrease either (Fig. S10B). Therefore, regulation of fetal liver mRNAs differs *in vitro* from *in vivo*. It should be noted that Cnot3 expression is lower in fetal hepatocytes from Cnot3LKO than those from control mice after being cultured for 1 day in the differentiation medium. After 3- or 5-day culture in the differentiation medium, Cnot3 levels further decreased in fetal hepatocytes from Cnot3LKO mice as Alb levels increased (Fig. S10B), supporting the idea that Cnot3 suppression is incomplete at E16.5 (Fig. 1C). It is possible that incomplete Cnot3 suppression at the beginning resulted in comparable differentiation to control. We compared gene expression profiles and mRNA half-lives in the differentiated fetal hepatocytes at d5, when Cnot3 was efficiently suppressed. Around 1400 mRNAs were upregulated more than 1.5-fold and 800 were downregulated more than 1.5-fold in the d5 *Cnot3^−/−^* fetal hepatocytes (Fig. S10C). Lists of differently expressed mRNAs partly overlapped between d3 livers and d5 fetal hepatocytes (Fig. S10D). We found that around 3000 mRNAs had longer half-lives (>2-fold) in *Cnot3*^−/−^ fetal hepatocytes (Fig. S10E and Table S4). Importantly, enriched GO terms of stabilized mRNAs included cell cycle and cell division, as in the case of 4-week-old *Cnot3^−/−^*liver, partly reflecting the phenotypes (Fig. S10F and Table S5). We confirmed stabilization of several mRNAs in *Cnot3*^−/−^ fetal hepatocytes by time course analysis of mRNA expression levels following actinomycin D (ActD) treatment (Fig. S10G). Fetal liver mRNAs hardly decreased after ActD treatment in either genotype, further suggesting different regulation of those mRNAs *in vitro* and *in vivo*.

### Cnot3 suppression leads to stabilization and poly(A) tail elongation of mRNAs for cell cycle-related and fetal liver genes, and transcriptional downregulation of those responsible for mature liver function

**Fig. 7.**
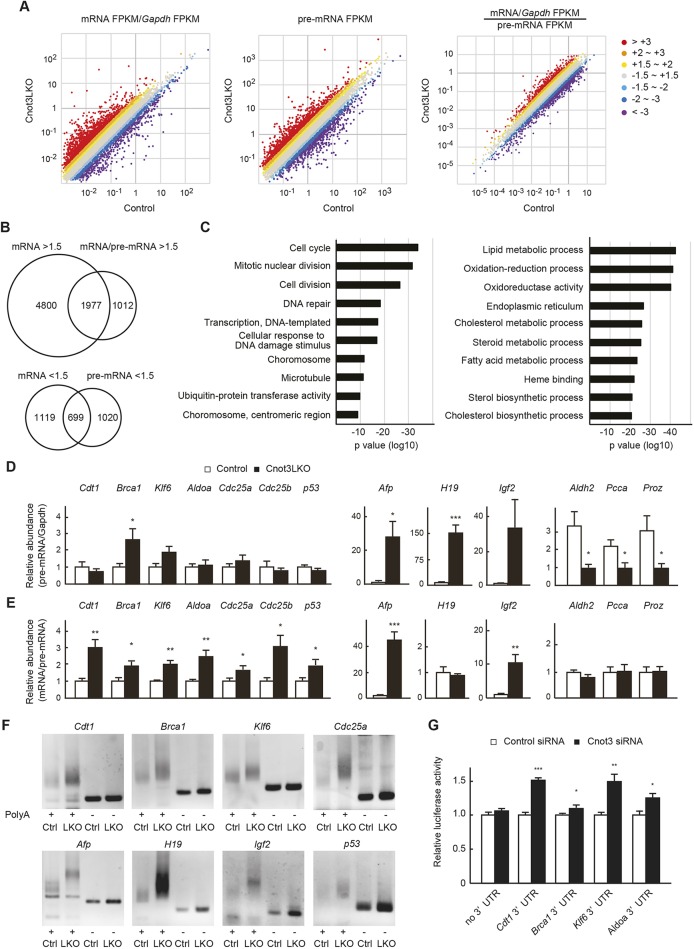
**Upregulated genes in *Cnot3*^−/−^ livers demonstrate mRNA stabilization and elongated poly(A) tails, whereas downregulated genes demonstrate decreased transcription.** (A) Scatter plots of *Gapdh*-normalized mRNA (left), pre-mRNA levels (middle) and mRNA/pre-mRNA ratios (right) in 4-week-old (4 w) control and *Cnot3*^−/−^ livers (*n*=3). (B) Venn diagrams illustrating the overlap of genes showing mRNA upregulation and stabilization >1.5-fold (upper), and those showing both mRNA and pre-mRNA downregulation >1.5-fold (lower) in *Cnot3*^−/−^ livers. (C) The results of GO analysis of the overlapping genes (left from the upper Venn diagram in B, right from the lower) are shown as bar charts of the top 10 GO terms ranked by *P* value. Gene lists included in each GO term are summarized in Table S5. (D,E) qPCR analysis of pre-mRNA and mRNAs in 4 w control and *Cnot3*^−/−^ livers. Graphs show relative abundances of pre-mRNAs (D) and mRNA/pre-mRNA ratios (E) of the indicated genes (*n*=5-7). (F) Comparison of poly(A) tail lengths of mRNAs in 4 w control (Ctrl) and *Cnot3*^−/−^ livers (LKO). Total RNA was subjected to PCR-based analysis (see Materials and Methods). PCR products without poly(A) regions (-) were also loaded. Similar results were obtained with two different littermates. (G) Luciferase assay with reporter constructs harboring the indicated 3′UTRs in Hepa1-6 cells transfected with control or Cnot3-targeting siRNA (*n*=3). Luciferase activity of each construct in control siRNA-transfected cells is set to 1. We performed five independent experiments. Data are mean±s.e.m. *P<0.05; **P<0.01; ***P<0.001.

To further characterize upregulated and downregulated mRNAs in Cnot3LKO mice, we performed RNA sequencing (RNA-seq). Because unspliced premature mRNAs (pre-mRNAs) are rapidly processed after transcription (Singh and Padgett, 2009; Oesterreich et al., 2011), their RNA-seq profiles have been shown to provide good proxies for transcription (Koike et al., 2012; Du et al., 2014). Compared with the microarray analysis, we detected many more upregulated and fewer downregulated mRNAs in *Cnot3^−/−^* livers (Fig. 7A). Importantly, upregulated mRNAs largely overlapped with those detected in microarray analyses (Fig. S11). A number of upregulated and downregulated pre-mRNAs indicated that Cnot3 suppression induced both transcriptional activation and suppression in liver (Fig. 7A). As an estimate of mRNA stability, we calculated mRNA to pre-mRNA ratios (Du et al., 2014). More than 65% (1977 of 2989 mRNAs) of stabilized mRNAs (>1.5-fold increase in mRNA/pre-mRNA ratios) were among the upregulated mRNAs (>1.5-fold increase in mRNA levels), suggesting that mRNA stabilization effectively contributed to upregulation of mRNAs in *Cnot3^−/−^* livers (Fig. 7B). Those 1977 mRNAs are involved in cell cycle, mitotic nuclear division and cell division (Fig. 7C, left and Table S6). mRNAs encoding molecules involved in cell-cell interaction and immunoresponses were mainly upregulated by transcriptional activation because they displayed upregulation of both mRNAs and pre-mRNAs (Fig. S12). We also detected mRNAs that underwent both transcriptional upregulation and stabilization, such as *Afp*. In contrast, mRNAs relevant to liver functions, such as lipid metabolism, oxidation-reduction and cholesterol metabolism, displayed downregulation of both mRNAs and pre-mRNAs (Fig. 7C, right and Table S6). Therefore, a transcription of the mature liver mRNAs was less activated in *Cnot3^−/−^* livers than in controls. These data were confirmed by analyzing pre-mRNA levels and mRNA/pre-mRNA ratios using qPCR (Fig. 7D,E). Finally, to clarify whether stabilization of mRNAs in *Cnot3^−/−^* livers was relevant to dysregulated deadenylation, we analyzed poly(A) tail lengths of *Cdt1*, *Brca1*, *Klf6*, *Afp*, *H19*, *Igf2*, *Cdc25a* and *Trp53* mRNAs. Their poly(A) tails were longer in *Cnot3^−/−^* livers than in controls (Fig. 7F). To investigate mechanisms by which Cnot3 regulates stability of *Cdt1*, *Brca1*, *Klf6* and *Aldoa* mRNAs, we isolated and inserted their 3′UTRs into a luciferase reporter plasmid. Reporter assays using Hepa1-6 cells, a murine hepatoma cell line, showed that Cnot3 regulates amounts of reporter genes in a manner dependent on each 3′UTR (Fig. 7G).

## DISCUSSION

The liver serves various functions to maintain organismal homeostasis. A complex, but well-organized network of transcription factors regulates liver development and function. During liver development, while genes encoding enzymes for metabolism or detoxification are induced, fetal liver-specific genes are silenced as hepatocytes mature (Spear et al., 2006). Cnot3LKO mice, which cannot deadenylate mRNAs in the liver, show various abnormalities, such as hepatocyte death, inflammation and anemia, coincident with stabilization of various genes. These phenotypes are very different from those observed in *Cnot3^+/−^* mice (Morita et al., 2011). *Cnot3^−/−^* livers show decreased expression of several metabolic genes essential for tissue function, indicating critical involvement of Cnot complex-mediated gene silencing in liver functional maturation.

Suppression of Cnot3 mediated by Alb-cre was not sufficient in E16.5 livers, but Cnot3 was efficiently suppressed in liver at d3 or later (Fig. 1C and Fig. S1). *Cnot3^+/−^* mice with reduced Cnot3 expression show normal liver histology, except for liver mass (Morita et al., 2011), suggesting that around 50% reduction in Cnot3 has no effect on fetal liver function. Thus, our results define the roles of Cnot3 and deadenylation in postnatal liver functional maturation. Because the Cnot complex is involved in early development, such as embryogenesis and ES differentiation (Hu et al., 2009; Morita et al., 2011), it is conceivable that the Cnot complex regulates fetal liver specification, development and function. Use of Cre expression constructs driven by *Afp* promoter or other earlier development promoters will help to clarify those roles.

**Fig. 8.**
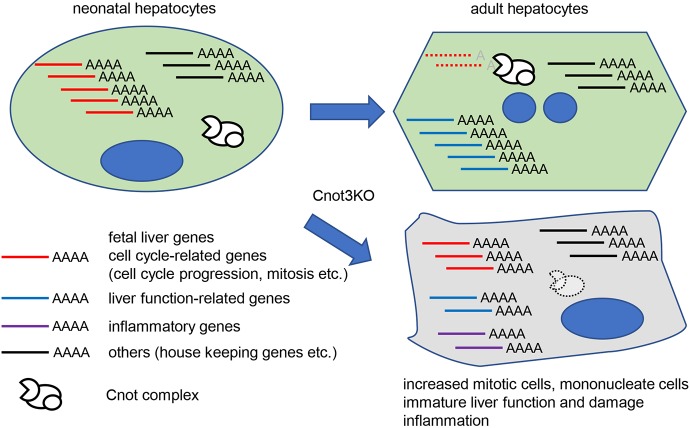
**A schematic model of roles of Cnot3-mediated deadenylation in postnatal liver functional maturation.** Expression of cell cycle-related and fetal liver mRNAs decreases as the liver matures. Cnot3-mediated deadenylation contributes to downregulation of those mRNAs during postnatal days. Molecules from several unusually stabilized mRNAs, which are involved in cell cycle progression and cytokinesis, lead to an increase of mitotic and mononucleate hepatocytes in *Cnot3^−/−^* livers. Transcription of mature liver genes is insufficient in *Cnot3^−/−^* livers. This is partly due to antagonistic effects of cell cycle-related molecules, such as Cdk activators and p53, on transcription factors responsible for liver maturation. Decreased expression of mature liver mRNAs is relevant to impaired liver function and damage.

Suppression of any subunits of the Cnot complex in cell lines results in stabilization of cell cycle-related genes due to elongation of poly(A) (Morris et al., 2005; Morita et al., 2007; Aslam et al., 2009; Mittal et al., 2011; Takahashi et al., 2012; Suzuki et al., 2015). Tissue-specific suppression of Cnot3 in mice had similar effects (Inoue et al., 2015; Li et al., 2017 and this study). mRNA abundance is regulated by the Cnot complex in a 3′UTR-dependent manner. These data indicate that regulating stability of cell cycle-related genes is one of the fundamental roles of the Cnot complex, though target mRNAs appear to vary depending on cell types. When liver development is complete, most hepatocytes enter a comparatively quiescent state. Expression of cell cycle progression-related mRNAs decreases concomitantly as liver matures. mRNAs encoding molecules responsible for the cell cycle checkpoint show similar expression changes, because the cell cycle checkpoint is generally required in proliferating cells, but not in quiescent cells to ensure proper cell cycle progression. Indeed, various cell cycle-related mRNAs, including both growth-promoting genes and cell cycle checkpoint genes, are more highly expressed in fetal liver than in adult liver (Li et al., 2012). Our data indicate that postnatal liver functional maturation also involves a decrease of cell cycle-related mRNAs (Fig. S9). Based on the data, we propose that the Cnot complex contributes to developmental stage-dependent proliferation cessation in liver from birth to adulthood by destabilizing cell cycle-related mRNAs (Fig. 8). Liver is composed of mononucleate and binucleate hepatocytes and binucleate hepatocytes emerge at about 1-2 weeks of age and increase in abundance during later stages in mice (Hsu et al., 2016). Significantly increased number of mononucleate diploid hepatocytes in *Cnot3*^−/−^ livers, concomitant with upregulation of mRNAs for cytokinesis promoting molecules, further suggested the functional immaturity of *Cnot3*^−/−^ livers (Figs 2 and 5). Cell proliferation and differentiation have mutually antagonistic effects in various cell types. For example, direct antagonism between cell cycle-promoting molecules, such as CDK/cyclin complexes and tissue-specific transcription factors, has been reported (Berasain and Avila, 2015; Ruijtenberg and van den Heuvel, 2016). Moreover, p53 represses the transcriptional function of HNF4α (Maeda et al., 2002), which plays a role in postnatal hepatocyte differentiation (Hayhurst et al., 2001). Downregulation of mature liver genes in *Cnot3^−/−^* livers was concomitant with decreased expression of pre-mRNAs, indicating that those genes are not efficiently transcribed (Fig. 7). It is possible that an increase of cell cycle-related mRNAs such as Cdk-activators and p53 would be, at least in part, involved in the insufficient transcription.

We detected elongated poly(A) tails and increased mRNA/pre-mRNA ratios of *Afp* and *Igf2* gene in *Cnot3*^−/−^ livers, suggesting potential involvement of post-transcriptional mechanisms in regulating an abundance of fetal liver mRNAs (Fig. 7). Transcription regulation largely involves developmental repression of *Afp* and *H19* genes (Tilghman and Belayew, 1982; Long and Spear, 2004; Nguyen et al., 2005; Xie et al., 2008). On the other hand, several studies have suggested a post-transcriptional mechanism in postnatal repression of *Afp* (Vacher et al., 1992; Peterson et al., 2011). It is tempting to speculate that increased expression of fetal liver genes in postnatal *Cnot3*^−/−^ livers is relevant to insufficient reduction of the mRNAs (Fig. 8). We cannot exclude the possibility that the Cnot complex regulates their transcription. Indeed, the Cnot complex is also responsible for transcriptional regulation in mammalian cells (Nakamura et al., 2004; Hu et al., 2009; Neely et al., 2010). The increase of *H19* RNA is mainly caused by the pre-mRNA increase (Fig. 7D), and the Afp protein was hardly detected in 8-week-old *Cnot3*^−/−^ livers despite a significant increase in its mRNA (Fig. 5G). Further analyses are necessary to address how transcriptional and post-transcriptional mechanisms mediated by the Cnot complex regulate fetal liver mRNAs during liver functional maturation.

Proper lipid metabolism in the liver requires mechanisms for post-transcriptional gene silencing, as mice lacking *miR-122* or *Dicer* develop hepatic steatosis (Sekine et al., 2009; Hsu et al., 2012; Tsai et al., 2012). Our microarray and subsequent GO analysis showed that lipid metabolic processes in livers of Cnot3LKO mice are downregulated (Fig. 4). Indeed, livers of 1 w Cnot3LKO mice showed prominent lipid accumulation (Fig. 1G). However, we did not detect ectopic lipid accumulation at later developmental stages, i.e. at 4 weeks. Livers of 1-week-old Cnot3LKO mice were still maturing, as mRNAs encoding some lipid metabolic enzymes were significantly upregulated between 1 and 4 weeks of age (Fig. 6B). The increase of lipid metabolism-related mRNAs might partly explain a decrease of lipid accumulation in 4-week-old *Cnot3*^−/−^ livers. These data suggested that, in the absence of Cnot3, delayed or impaired cell fate transition from fetal to adult may involve lipid accumulation.

We detected an increase of CK19-positive cells in 4-week-old *Cnot3^−/−^* livers (Fig. 2B). It is possible that ductular reaction contributes to the recovery of normal hepatocytes, because the reaction is relevant to differentiation from non-hepatic to hepatic cells and may have a role in liver regeneration (Sato et al., 2018). While 4- and 8-week-old *Cnot3^−/−^* livers displayed various abnormalities such as irregular hepatocyte alignment, necrosis, inflammation and anemia (Figs 1 and 3), our preliminarily studies detected milder liver pathology and appearance of Cnot3-expressing hepatocytes at later stages (12-16 weeks of age). How Cnot3-expressing hepatocytes appear and whether they contribute to phenotype recovery needs to be addressed in future work.

When the Cnot complex regulates stability of an mRNA, one possible molecular mechanism is miRNA/Ago2-dependent recognition of the targets (Zekri et al., 2009; Chekulaeva et al., 2011; Fabian et al., 2011; Huntzinger et al., 2013). Mice lacking *miR-122* or *Dicer* in liver share with Cnot3LKO mice several phenotypic abnormalities, such as lipid accumulation, increase of binucleate hepatocytes and hepatitis, and they also share specific changes in gene expression (Sekine et al., 2009; Hsu et al., 2012; Tsai et al., 2012; Hsu et al., 2016). It should be noted that *Aldoa*, *Klf6* and *Iqgap1* mRNAs are the defined *miR-122* targets. B-cells and adipose tissues lacking *Cnot3* show similar phenotypes to those lacking *Dicer*, further supporting a functional relationship between the Cnot complex and miRNA in tissue development and function (Koralov et al., 2008; Mori et al., 2014; Inoue et al., 2015; Li et al., 2017). However, compared with mice lacking *miR-122* or *Dicer* in liver, Cnot3LKO mice showed more severe liver damage, as indicated by serum profiles, and they also manifest highly abnormal hepatocyte morphology. The number of upregulated genes in *Cnot3*^−/−^ livers (a few thousand genes, >2.0) was much greater than in *miR-122*KO mice (a few hundred genes, >2.0; Hsu et al., 2012; Tsai et al., 2012). These results are likely due to the fact that the Cnot complex uses various mRNA-binding proteins to recognize its targets (Chang et al., 2004; Chicoine et al., 2007; Fabian et al., 2013; Adachi et al., 2014; Yamaji et al., 2017). AREs and GU-rich elements (GREs) are well-characterized sequences that influence mRNA stability (Garneau et al., 2007; Vlasova-St. Louis and Bohjanen, 2011). Those sequences are frequently observed in 3′UTRs. When we searched for genes possessing AREs or GREs among upregulated genes in *Cnot3*^−/−^ liver using an ARE-containing mRNA DATABASE (Bakheet et al., 2006), some mRNAs, such as *pdk4* and *igfbp1*, which have already been identified as Cnot complex targets (Morita et al., 2011), contained these sequences in their 3′UTRs; however, *Brca1* and *Cdt1* mRNAs did not. Therefore, the Cnot complex regulates levels of *Brca1*, *Cdt1* and many other mRNAs through sequences in their 3′UTRs that are distinct from AREs and GREs.

In summary, our study describes tissue-specific roles of mRNA decay involving deadenylation. Suppression of Cnot3 in liver affects expression of mRNAs encoding developmentally regulated and cell cycle-related molecules. Those genes sharply decline in liver at about the time of weaning. Therefore, Cnot3LKO mice illustrate a plausible mechanism by which liver gene expression profiles shift during development from the postnatal stage to adulthood, manifesting the role of mRNA decay in developmental stage progression, which involves dramatic changes in gene expression.

## MATERIALS AND METHODS

### Generation of Cnot3LKO mice

Mice carrying the floxed allele of *Cnot3* (Inoue et al., 2015; Li et al., 2017) were crossed with Alb-cre and mTmG mice (Jackson Laboratory, #003574 and #007676, respectively). Experiments were performed according to animal use guidelines issued by the Committee of Animal Experiments at Okinawa Institute of Science and Technology Graduate University and the Institutional Animal Care and Use Committee of RIKEN Yokohama Branch. We used mice possessing genotypes (flox/+), (flox/flox), Alb-cre;(+/+) or Alb-cre;(flox/+) as controls, because mice with those genotypes were indistinguishable in appearance, histology and serology from wild type. In addition, both male and female Cnot3LKO mice showed similar phenotypes, which we describe in this report.

### Blood tests and biochemical examination of blood

Blood or plasma for each analysis was obtained by cardiac puncture from deeply anesthetized mice following overnight fasting (only for plasma), and was analyzed by the Oriental Yeast Company (Japan).

### Cell culture

Hepa1-6 cells (RCB1638) were from RIKEN Bioresource Center and were used within 10 passages. They were cultured at 37°C in Dulbecco's modified Eagle's medium (DMEM) (Wako) supplemented with 10% fetal bovine serum (FBS). Fetal hepatocytes were cultured in DMEM containing 10% FBS, non-essential amino acids (Gibco, 11140-050), ITS (Gibco, 41400-045), 50 µg/ml gentamycin (Sigma), 10 ng/ml oncostatin M (R&D systems) and 100 nM dexamethasone (Sigma). To prepare fetal hepatocytes, embryonic liver (E16.5) was minced and incubated with liver perfusion medium (Gibco 17701-038) at 37°C for 7 min. Liver fragments were pelleted by centrifugation (100 ***g***, 800 rpm for 3 min) and incubated with liver digest medium (Gibco 17703-034) following removal of liver perfusion buffer at 37°C for 15 min. After pipetting to dissociate cells completely, the cell solution was filtered with a 70 µm cell strainer and centrifuged (200 ***g***, 1200 rpm for 3 min). Cell pellets were suspended with hemolysis solution [16.5 mM Tris-HCl, 0.56% ammonium chloride (pH 8.0)] and incubated on ice for 7 min. Cells were pelleted by centrifugation (200 ***g***, 1200 rpm for 3 min) following addition of fetal hepatocyte medium. Cell pellets were re-suspended in medium and plated onto 0.1% gelatin-coated dishes. Four hours later, floating non-hepatic cells and cell debris were removed by extensive washing. We changed the culture medium every 2 days. We used cells for experiments 5 days after preparation. For isolation of primary hepatocytes, adult mice (4-5 weeks) were subjected to a collagenase perfusion. The liver was perfused with collagenase solution, 18 mM Hepes-NaOH (pH 7.4), 0.075% NaHCO_3_, 0.5 µg/ml insulin, 0.1 mg/ml collagenase (Sigma, C2674) in 1× Hank's solution, through the portal vein. Perfused hepatocytes were washed with PBS three times and fixed with ethanol.

### FACS analysis of hepatocytes

Ethanol-fixed hepatocytes were washed with PBS and incubated with 0.5 mg/ml RNase A (Sigma, R4875) for 30 min at 37°C. Cells were then stained with propidium iodide (Wako, 169-26281) and subjected to flowcytometry using a FACS Calibur (BD Biosciences). We used FlowJo V10.4.2 (BD Biosciences) to analyze the results.

### Antibodies and reagents

Antibodies against Cnot1, Cnot2, Cnot6L, Cnot7, Cnot8 and Cnot9 have been described previously (Suzuki et al., 2015). Antibodies against α-tubulin (DM1A, sc-32293), Cdt1 (sc-365305), Brca1 (sc-135731), Alb (sc-46291, for WB), normal mouse IgG (sc-2025) and normal rabbit IgG (sc-2026) were purchased from Santa Cruz Biotechnology. Antibodies against cleaved caspase-3 (9664) and p53 (2524) were from Cell Signaling Technology. Anti-Ki67 rabbit monoclonal antibody for immunohistochemistry was from NeoMarkers (RM-9106-S0). Antibodies against CK19 (ab133496), Ki67 (ab16667), phospho-histone H3 (Ser10) (ab5176), Afp (ab46799), Igf2 (ab9574), CD45 (ab10558) and F4/80 (ab111101) were from Abcam. Anti-Alb antibody (MAB-1455, for immunofluorescence) was from B&D systems. Antibodies against Chk1 (K0086-3) and Iqgap1 (K0100-3) were from MBL. Anti-Klf6 antibody (14716-1-AP) was from Proteintech.

### Immunoprecipitation and immunoblotting

Liver or cells were lysed with lysis buffer [1% NP-40, 50 mM Tris-HCl (pH 7.5), 150 mM NaCl, 1 mM EDTA, 1 mM phenylmethylsulfonylfluoride and 10 mM NaF). Liver lysates (1 mg) were subjected to immunoprecipitation using anti-Cnot3 antibody (1 µg) and protein G sepharose 4 FF (GE Healthcare, 17-0618-01). We used 20 µg of lysates for immunoblotting. Immunoprecipitation and immunoblotting were performed as described previously (Suzuki et al., 2015). Anti-Igf2 and anti-Brca1 antibodies were used at 1:750 dilution, the other antibodies were used 1:1000 dilution.

### Histology, immunohistochemistry and immunofluorescence

Dissected livers were fixed with 10% formaldehyde for Hematoxylin and Eosin staining or 4% paraformaldehyde (PFA) in phosphate-buffered saline (PBS) for other procedures. Fixed livers were processed as paraffin-embedded sections or cryosections. We used paraffin-embedded sections for Hematoxylin and Eosin staining, Masson's trichrome staining and immunohistochemistry. Cryosections were employed for Oil Red-O followed by Hematoxylin staining, rhodamine-phalloidin staining (Molecular Probes) or mTmG reporter protein detection, together with 4′,6-diamidino-2-phenylindole (DAPI, 1 µg/ml, Sigma, D9564) staining. For antigen retrieval, glass slides were incubated in citrate buffer (pH 6.0) at 120°C for 15 min. After inactivating endogenous peroxidase with 3% H_2_O_2_ in methanol, slides were incubated with 5% normal goat serum (ThermoFisher, 16210-064) in phosphate-buffered saline for 30 min and subsequently incubated with primary antibodies (1:200 or 1:500 for Igf2) at 4°C overnight. For detection, Histofine Simple Stain Mouse MAX PO was used as the secondary antibody in combination with a DAB staining kit (Nichirei). Hematoxylin 3G (8656) and Eosin (8659) were from Sakura Finetek Japan. For immunofluorescence, we carried out a transcardial perfusion with 4% PFA and picked up livers from the fixed mice. Livers were further fixed with 4% PFA at 4°C overnight. We incubated fixed livers with 10% and 30% sucrose for 5 min and more than 6 h, respectively. We prepared 6 µm frozen sections. Dried sections on slide glasses were washed with PBS three times and incubated with blocking buffer (5% normal goat serum, 0.1% Triton X-100 in PBS) for 1 h and subsequently incubated with primary antibodies (1:100 dilution) at 4°C overnight. Slides were washed with PBS three times, and incubated with secondary antibodies, goat anti-rabbit IgG Alexa Fluor 488 and goat anti-mouse IgG Alexa Fluor 546 (ThermoFisher, A11008 and A11030, 1:1000 dilution), and DAPI for 1 h. After washing with PBS, Fluoromount (Diagnostic Biosystems, K024) was used for mounting. We captured images and counted the number of stained cells (antibodies and Hematoxylin) using a BZ X-700 microscope (Keyence).

### Microarray analysis

Total RNA was extracted with ISOGEN II according to the manufacturer's protocol (Nippon Gene). RNA quality was determined using the Bioanalyzer Nano Chip (Agilent Technologies). cDNA and biotin-labeled cRNA were synthesized according to the manufacturer's instructions (Affymetrix). After fragmentation of cRNA, biotin-labeled cRNA was hybridized to the GeneChip Mouse Genome 430 2.0 Array (Affymetrix). To determine the average difference for each probe set, we used the Robust Multi-array Average algorithm. Selected probeset IDs were converted according to the manufacturer's instructions. To determine differences in gene expression and to compare gene expression profiles among data sets, we used GeneSpring 14.9 (Agilent Technologies). We filtered probe sets by expression (lower cut-off, 20%) and used them to create scatter plots and carry out other analyses. The complete data set reported herein has been submitted to the NCBI Gene Expression Omnibus (GEO) database (www.ncbi.nlm.nih.gov/geo/) as a SuperSeries GSE114570 (GSE114507, GSE114508, GSE120910 and GSE 120911). Microarray data of *miR122*-deficient mice were obtained from the GEO database (GSE20610 and GSE27713). We used DAVID Bioinformatics Resources (david.ncifcrf.gov/) for gene ontology analysis.

### RNA-sequencing

Total RNA (1 µg) was used for RNA-seq library preparation with a TruSeq Stranded mRNA LT Sample Prep Kit (Illumina), which allows highly specific polyA-oligo(dT)-based purification of mRNA, according to the manufacturer's protocol. 109 bp pair-end read RNA-seq was performed using a Hiseq PE Rapid Cluster Kit v2-HS and a Hiseq Rapid SBS Kit v2-HS (200 Cycle) on a Hiseq2500 (Illumina), according to the manufacturer's protocol. For data analysis, using StrandNGS software (Strand Genomics) reads were mapped to the Ensembl genome sequence (mm10) and FPKMs (fragments per kilobase of exon per million mapped fragments) were calculated. Exonic FPKMs were normalized with *Gapdh* FPKM to calculate gene expression values. Genes with gene expression values <0.001 were excluded. Similar results were obtained using *Rplp0* FPKM for normalization. For calculating intronic FPKMs (pre-mRNA FPKMs), reads mapped to the intronic region were extracted with StrandNGS software, and read numbers were normalized by the total count of reads mapped to the intronic region and the sum of intron lengths. Genes with FPKMs <0.01 and <30 bp intron length were eliminated. We used total RNAs prepared from livers in Cnot1(flox/flox), Cnot1(flox/flox):Alb-Cre (Cnot1LKO) and Cnot3LKO mice. As Cnot1(flox/flox) mice are identical to wild-type mice (Yamaguchi et al., 2018; A.T. and T.Y., unpublished) and all the strains have the same genetic background (backcrossed with C57BL/6 eight times), we used them as controls in these analyses. A comparison of Cnot1 and Cnot3 roles in liver development will be reported elsewhere. Sequence data are available through ArrayExpress under the accession number E-MTAB-7182.

### Reporter assay

We amplified 3′UTRs (*Cdt1*, *Brca1*, *Klf6* and *Aldoa* mRNAs) by PCR using KOD Plus Neo (Toyobo) as the enzyme and 1-month-old mouse liver cDNA as a template, and inserted them into psiCHECK-1 Vector (Promega). We used a pRc/CMV-firefly luciferase construct (Fukao et al., 2014) as an internal control. Hepa1-6 cells (1.6×10^4^ cells) were transfected with 4.2 pmol of control small interfering RNA (siRNA) or siRNA against mouse Cnot3 using Lipofectamine RNAiMAX (ThermoFisher) on 48-well dishes. Forty-eight hours later, reporter constructs (250 pg each) and pcDNA3.1 (0.15 µg, ThermoFisher) were introduced into siRNA-transfected Hepa1-6 cells using lipofectamine 2000 (ThermoFisher). Twenty-four hours later, cells were lysed and subjected to reporter analysis using Dual-Luciferase Reporter Assay System and GloMax Discover System (Promega).

### RNA analysis

Total RNA (4 µg for *Igf2* pre-mRNA, 1 µg for the others) was used for reverse transcription with an oligo(dT)12-18 primer (Invitrogen) using the SuperScript III First-Strand Synthesis System (Invitrogen). qPCR reactions were carried out using SYBR Premix Ex Taq (Takara) and the ABI PRISM 7900HT Sequence Detection System (Applied Biosystems). *Gapdh* mRNA levels were used for normalization. To measure mRNA stability, cells were treated with ActD (2.5 µg/ml) for 6 or 12 h, and total RNAs were extracted at indicated times and subjected to qPCR analysis. Half-life was calculated as described previously (Chen et al., 2008). To compare poly(A) tail length, we used a Poly(A) Tail-Length Assay Kit (Affimetrix) according to the manufacturer's protocol. Primers for PCR reactions and poly(A) tail analyses are listed in Table S7.

### Statistical analyses

Differences between groups (control versus *Cnot3*^−/−^ or control siRNA versus Cnot3 siRNA) were tested for statistical significance using Student's *t*-test (unpaired, two-tailed distribution with two-sample equal variance). We considered a *P*<0.05 as statistically significant.

## Supplementary Material

Supplementary information
