# Supplementary material for: Postnatal liver functional maturation requires Cnot complex-mediated decay of mRNAs encoding cell cycle and immature liver genes
Source: Development. 2019 Feb 15;146(4):dev168146. doi: 10.1242/dev.168146 (PMC6398447; doi:10.1242/dev.168146)
Supplement: Supplementary information [file develop-146-168146-s1.pdf]

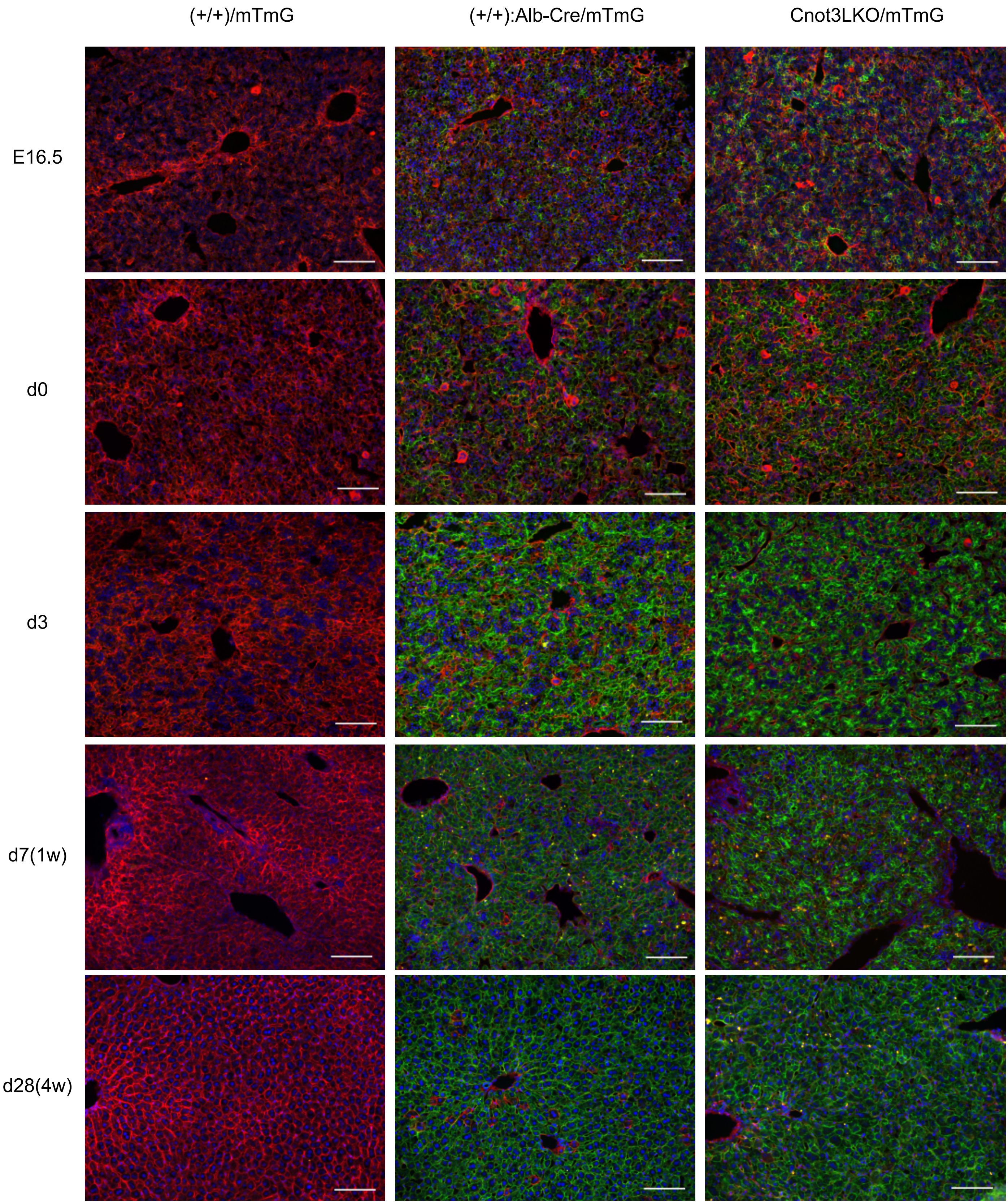

Figure S1. Albumin-cre-mediated recombination in livers at various developmental stages.
Frozen sections were prepared from livers of (+/+)/mTmG, (+/+):Alb-cre/mTmG and Cnot3LKO/mTmG mice at the indicated developmental stages. The sections were stained with DAPI (blue) and analyzed to detect fluorescent proteins (red; tdTomato, green; GFP) using microscope.
Scale bars, 100 μm

Development • Supplementary information

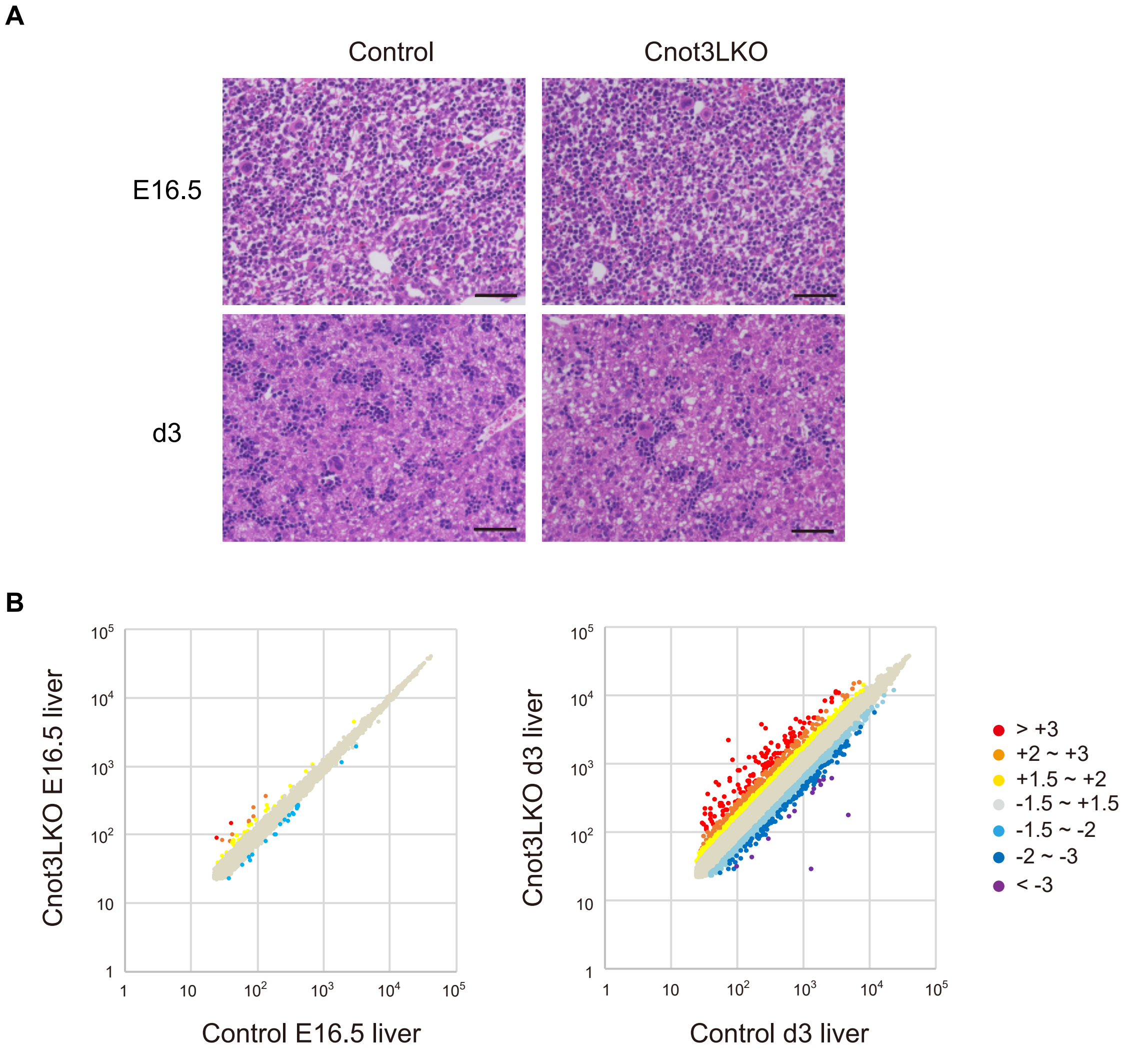

Figure S2. Differences in histology and gene expression appear in d3 *Cnot3*$^{-/-}$ livers.
(A) HE staining of livers from control and Cnot3LKO mice at E16.5 (upper panels) and d3 (lower panels). Scale bars, 50 µm.
(B) Scatter plots of mRNA expression depicted from the results of microarray using total RNAs from control or *Cnot3*$^{-/-}$ livers at E16.5 (left) and d3 (right) (*n*=2).

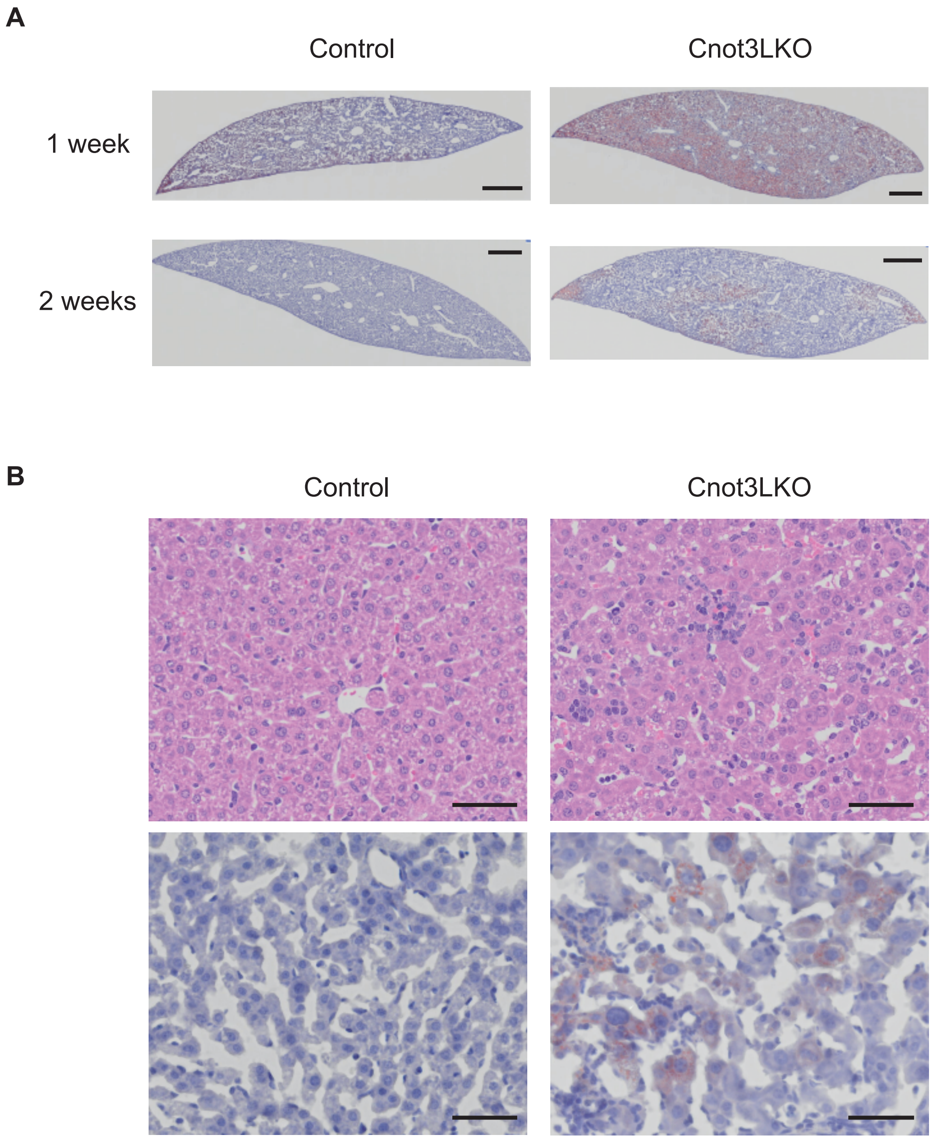

Figure S3. Lipid abnormally accumulates in *Cnot3*$^{-/-}$ livers at 1 week of age, but decreases at 2 weeks of age.(A) Oil red-O staining of livers from control and Cnot3LKO mice at 1 (upper) and 2 (lower) weeks of age. Scale bars, 500 μm.(B) Hematoxylin and Eosin (upper) and Oil red-O staining (lower) of livers from control and Cnot3LKO mice at 2 weeks of age. Scale bars, 50 μm.

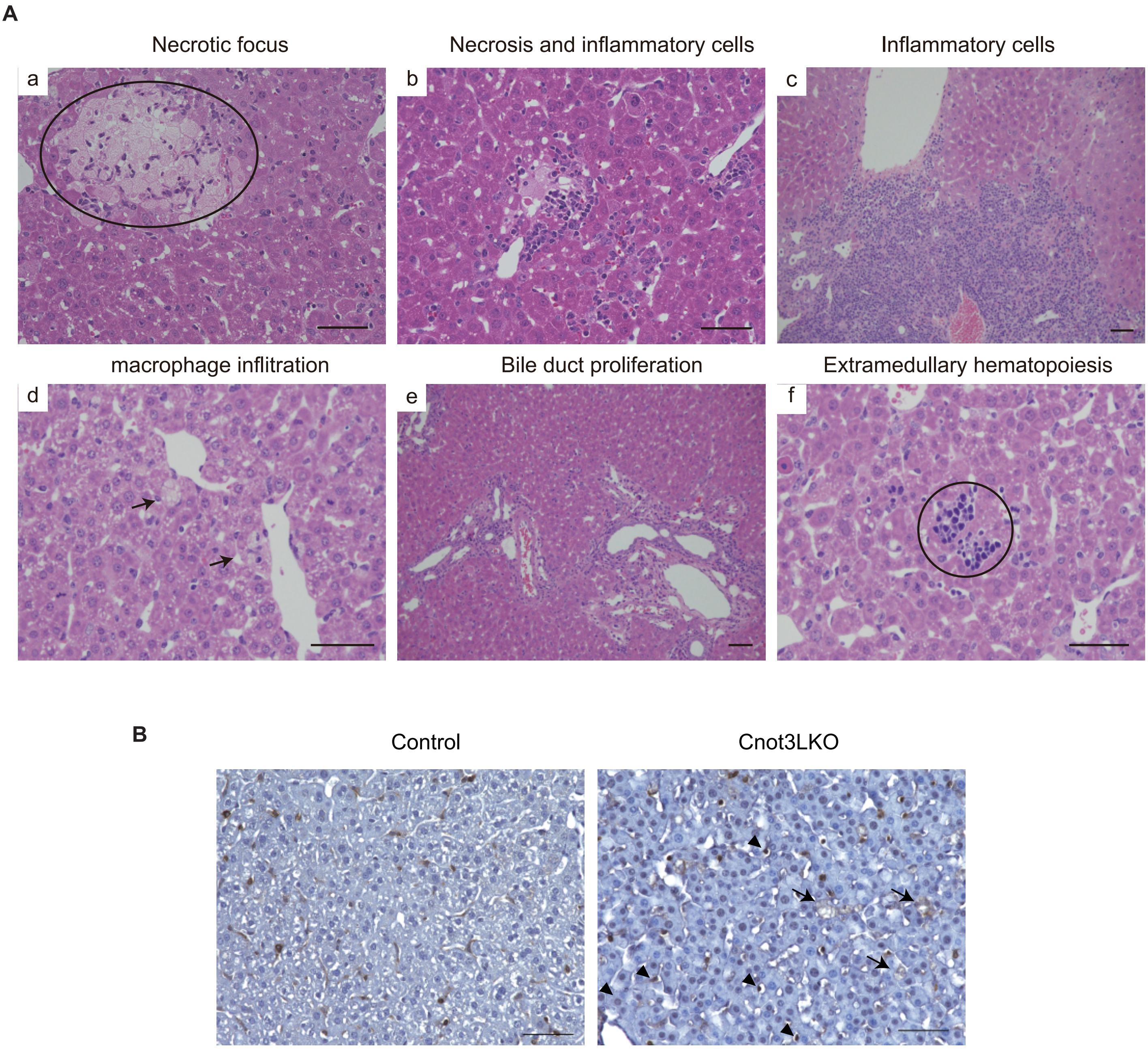

Figure S4. Abnormal histologies and infiltration of CD45-positive cells in *Cnot3*$^{-/-}$ liver.
(A) HE stained-*Cnot3*$^{-/-}$ livers at 4 weeks of age showing various pathology (a-f). Similar pathologies were observed at 8-week-old mice.
(B) Immunohistochemistry for CD45. Arrows and arrowheads indicate macrophages in the hepatocyte areas and liver resident macrophage,Kupffer cells, respectively. Scale bars, 50 μm.

Development • Supplementary information

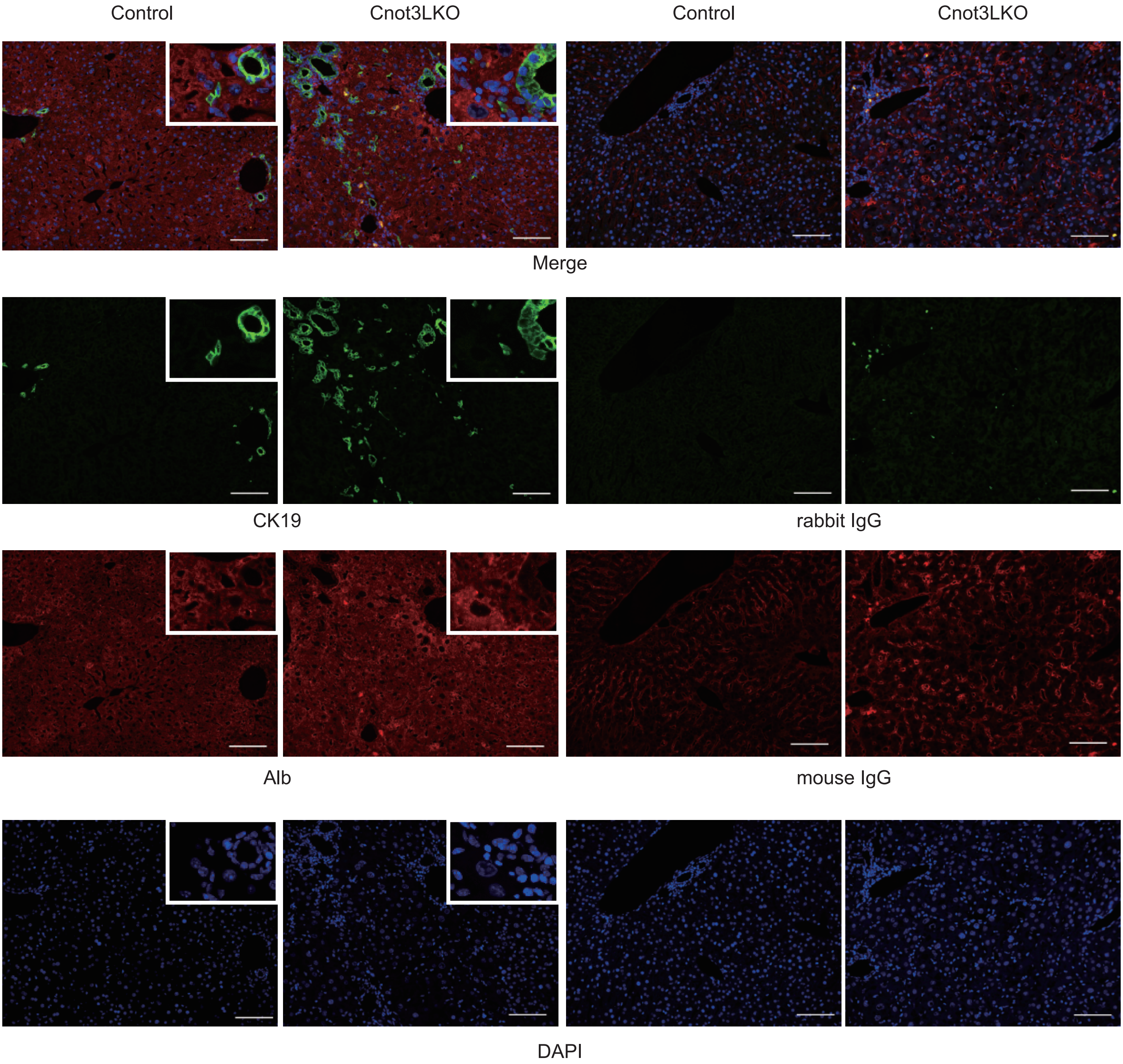

Figure S5. Enhanced biliary reaction in *Cnot3*$^{-/-}$ livers

Immunofluorescence of frozen liver sections from 4-week-old control and Cnot3LKO mice using mouse anti-Alb antibody and rabbit anti-CK19 antibody. Nuclei are stained with DAPI (bottom panels). Top panels show merged images. Normal rabbit and mouse IgGs are used as negative controls. Magnified views of representative areas (inset). Scale bars, 100 µm.

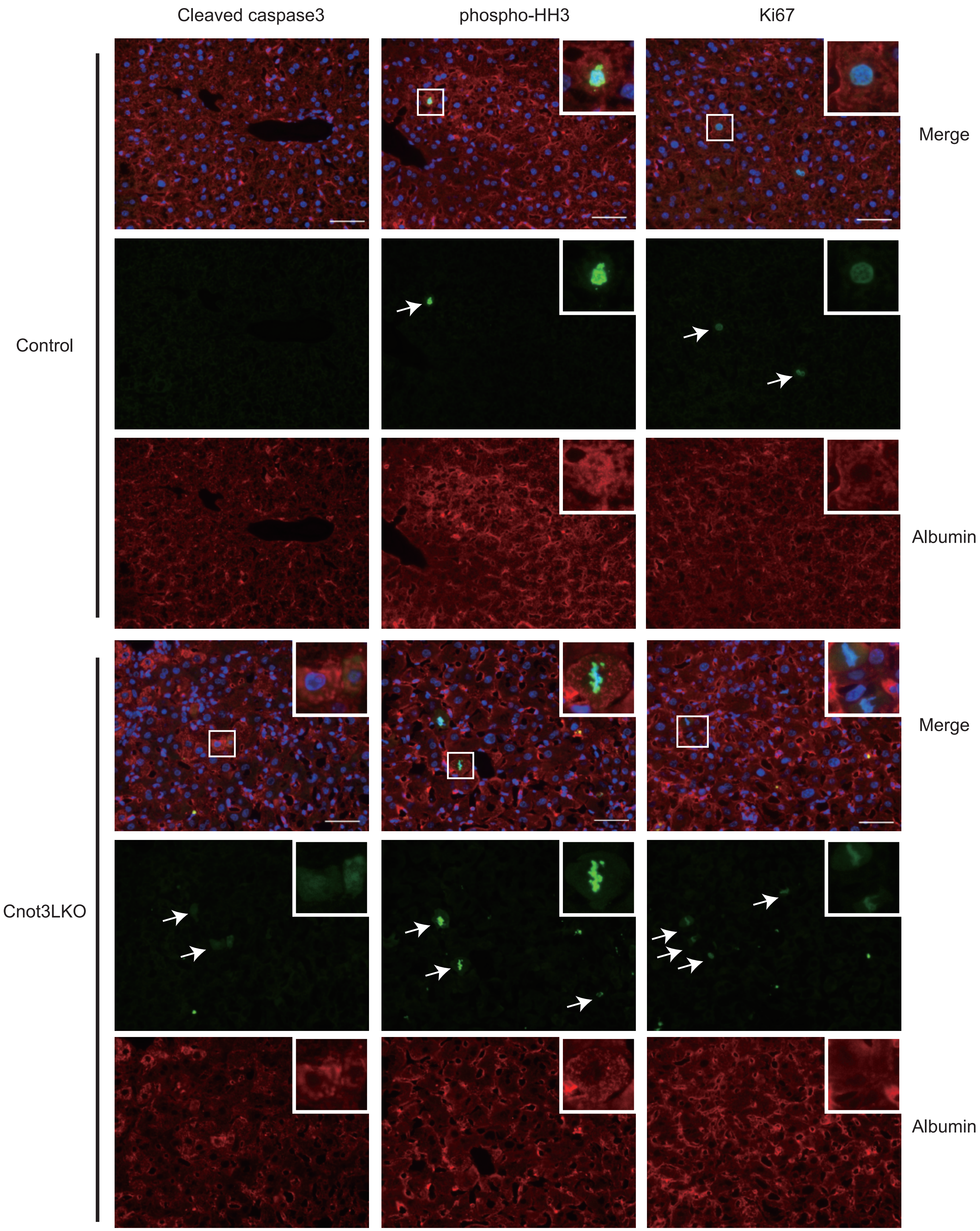

Figure S6. Enhanced apoptosis and mitosis in *Cnot3*$^{-/-}$ liver

Immunofluorescence of frozen liver sections from 4-week-old control (upper) and Cnot3LKO (lower) mice using indicated antibodies. Nuclei are stained with DAPI. Top panels in each genotype show merged images.

Magnified views of representative areas (inset). Scale bars, 100 μm.

Development • Supplementary information

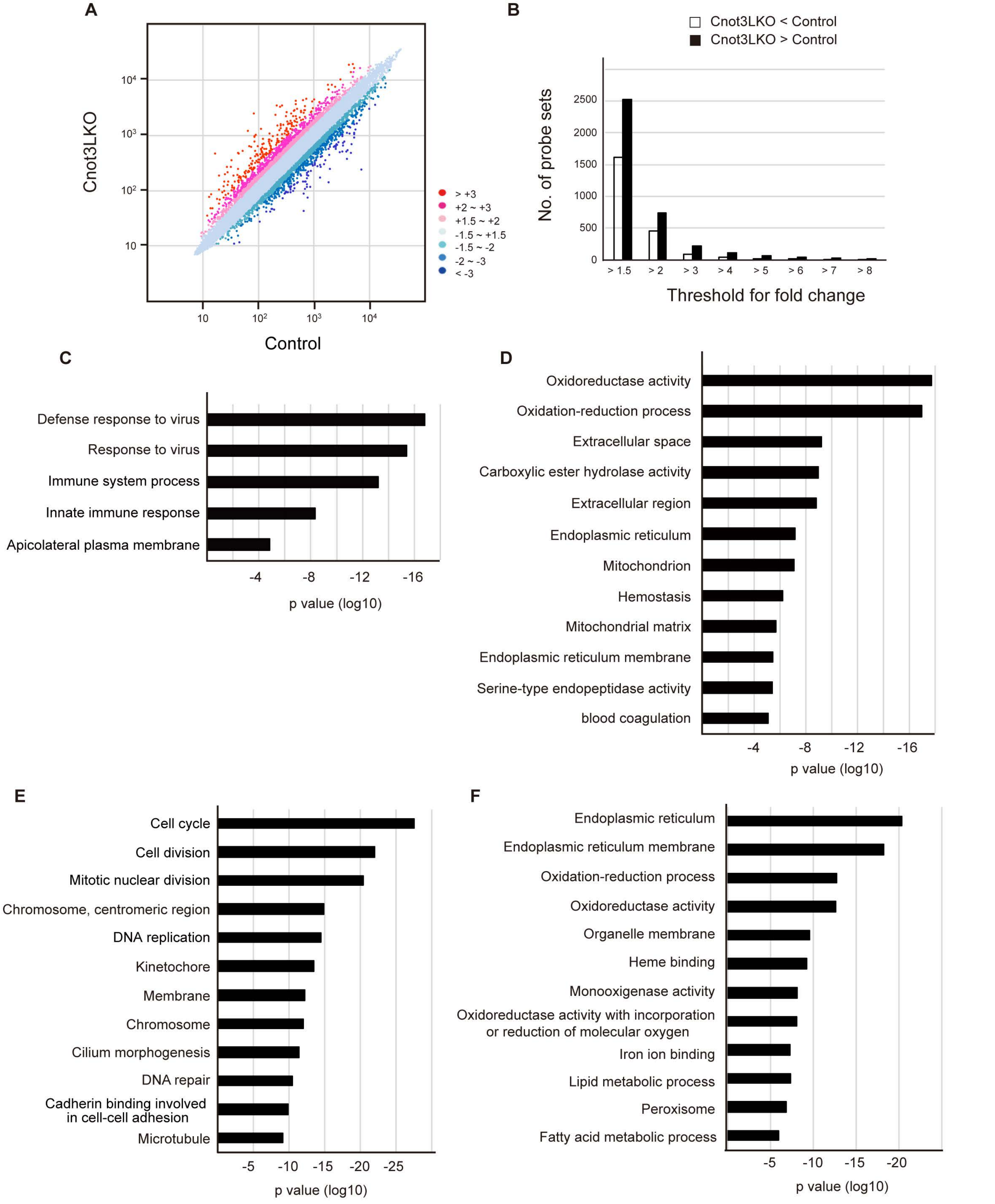

Figure S7. Gene expression and GO analysis in livers from 1-week-old Cnot3LKO mice
(A) Scatter plot of mRNA expression in livers from 1-week-old control and Cnot3LKO mice (*n*=4).
(B) Bar plot of the number of probe sets up-regulated (black bars) or down-regulated (open bars) with changes in *Cnot3*$^{-/-}$ livers relative to the controls.
(C, D) GO analysis of mRNAs upregulated (C) or downregulated (D) more than 2-fold in 1-week-old *Cnot3*$^{-/-}$ livers compared to the control.
(E, F) GO analysis of mRNA specifically upregulated (E) or down-regulated (F) at 4-week-old *Cnot3*$^{-/-}$ livers compared to 1-week-old.
Bar charts of the GO terms (FDR < 0.05, at most 12) ranked by P value are shown.
Gene lists included in each GO term (C,D) are summarized in Supplementary table 1.

Development • Supplementary information

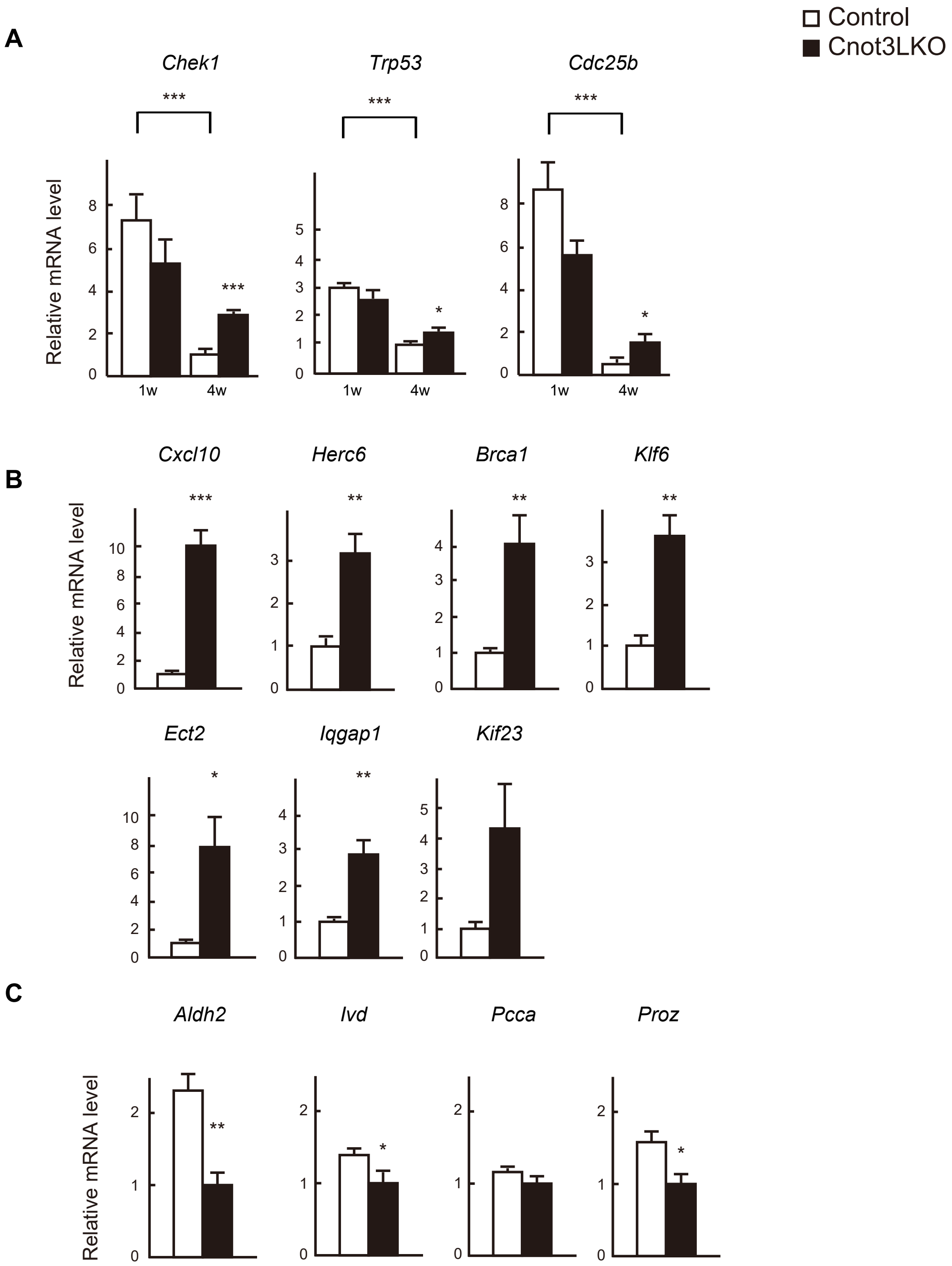

Figure S8. Increased inflammatory and cell cycle-related mRNAs and decreased liver function-related mRNAs in *Cnot3*$^{-/-}$ livers
(A-C) qPCR analysis of mRNAs in livers from 1, 4 (A) and 8-week-old (B, C) control and Cnot3LKO mice. *Gapdh* mRNA levels were used for normalization. Expression levels of genes in 4-week-old control mice (A), 8-week-old control mice (B) or Cnot3LKO mice (C) are set to 1 (*n*=6 for *Cdc25b*, *n*=5 for the others). Values in graphs represent means ± sem. *p < 0.05, **p < 0.01, ***p < 0.001

Development • Supplementary information

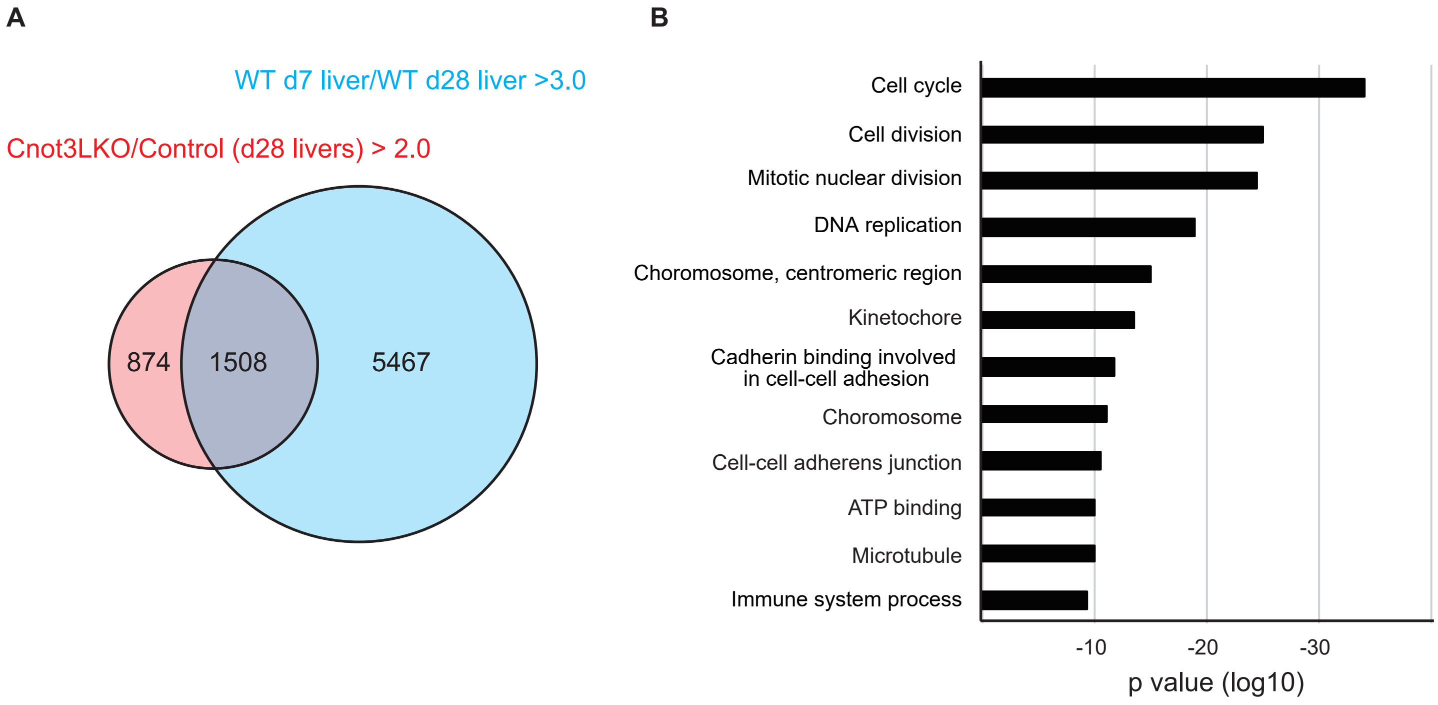

Figure S9. The upregulated mRNAs in *Cnot3*$^{-/-}$ livers are expressed at higher levels at 1 week of age than at 4 week age in wild-type livers. (A) Overlap of mRNAs upregulated in 4-week-old *Cnot3*$^{-/-}$ livers and those which decrease in wild-type livers from 1 to 4 weeks of age. Venn diagram of mRNAs that are expressed more than 2-fold higher in *Cnot3*$^{-/-}$ livers compared with controls at 4 weeks of age (red) and those that are expressed more than 3-fold higher in 1-week-old control livers compared with 4-week-old control livers (blue). *Gapdh*-normalized values are compared between 1-week-old and 4-week-old wild-type livers.
(B) GO analysis of the overlapped mRNAs in the Venn diagram (A). Bar charts of the GO terms (FDR < 0.05, at most 12) ranked by P value are shown. Gene lists included in each GO term are summarized in Supplementary table 3.

Development • Supplementary information

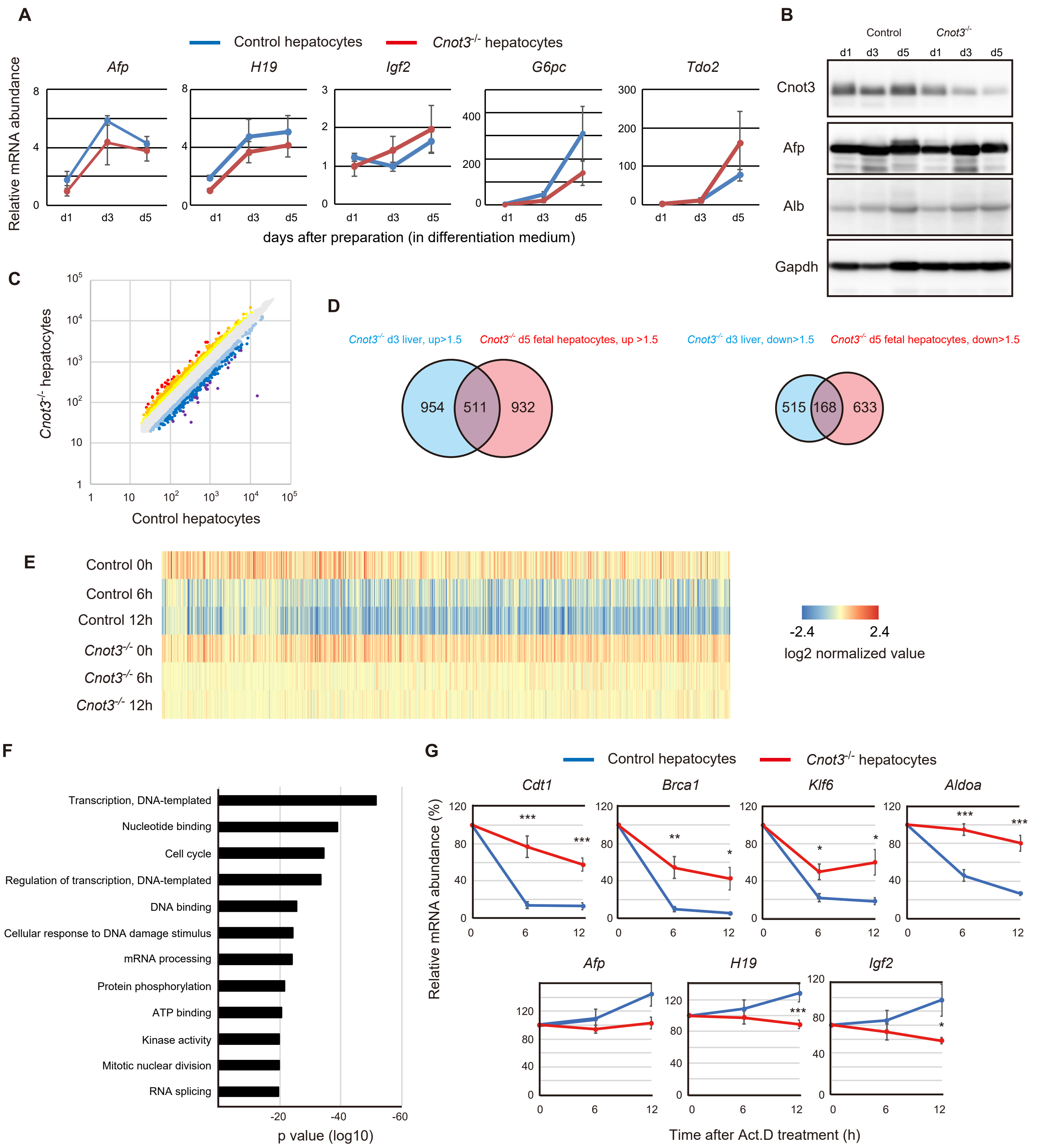

Figure S10. The *in vitro* differentiated Cnot3$^{-/-}$ fetal hepatocytes show stabilization of cell cycle-related mRNAs

(A, B) Control and *Cnot3*$^{-/-}$ fetal hepatocytes were cultured under differentiation condition. Total RNAs (A) or lysates (B) were prepared at the indicated period. Relative mRNA levels were determined by qPCR and normalized to *Gapdh* mRNA level (A). Expression levels of genes in *Cnot3*$^{-/-}$ fetal hepatocytes 1d after preparation are set to 1 (*n*=5). Values in graphs represent means ± sem. Immunoblots with the indicated antibodies are shown in (B).

(C) Scatter plot of mRNA expression in control and *Cnot3*$^{-/-}$ fetalhepatocytes at d5 (*n*=1).

(D) Comparison of diffently expressing mRNAs upregulated (left) or downregulated upon Cnot3 suppression between d3 livers and d5 fetal hepatocytes.

(E) Control and *Cnot3*$^{-/-}$ fetal hepatocytes were treated with Act. D at d5. Heat map of mRNAs having 2-fold longer half-lives in *Cnot3*$^{-/-}$ fetal hepatocytes. Gene lists are in Supplementary table 4.

(F) GO analysis of mRNAs having 2-fold longer half-lives in *Cnot3*$^{-/-}$ fetal hepatocytes. Bar charts of the top 12 GO terms ranked by P value are shown. Gene lists are in Supplementary table 5.

(G) Relative mRNA levels were determined by qPCR and normalized to the *gapdh* mRNA level. mRNA level without Act. D treatment (0 h) was set to 100% (*n*=5). *p < 0.05, **p < 0.01, ***p < 0.001

Development • Supplementary information

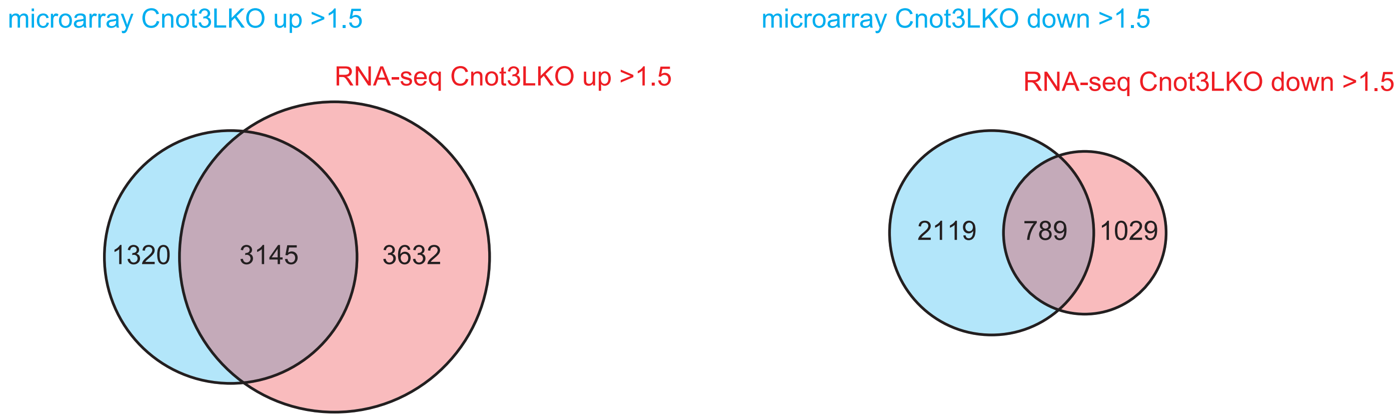

Figure S11. Overlap of differently expressing mRNAs in *Cnot3*$^{-/-}$ livers compared to control between microarray and RNA-seq. Venn diagrams of mRNAs which express higher (left) or lower (right) more than 1.5-fold in *Cnot3*$^{-/-}$ livers obtained from microarray (blue) or RNA-seq (red) analyses are shown.

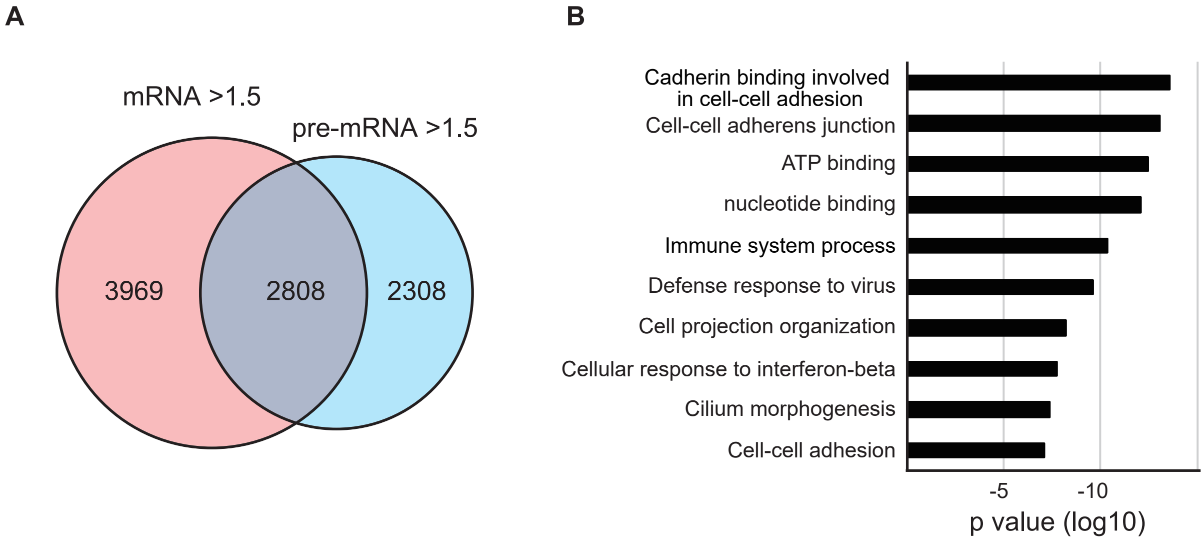

Figure S12. mRNAs relevant to cell-cell interaction and immunoresponces show both mRNA and pre-mRNA upregulation in *Cnot3*$^{-/-}$ livers.(A) Venn diagrams illustrating the overlap of genes showing mRNA (red) and pre-mRNA (blue) upregulation >1.5 fold in 4w *Cnot3*$^{-/-}$ livers calculated using the results of total RNA-seq.
(B) The result of GO analysis of the overlapping genes is shown as bar charts of the top 10 GO terms ranked by P value (right).
Gene lists included in each GO term are summarized in Supplementary table 6.

Development • Supplementary information

Table S1. GO analysis of differently expressing mRNAs in 1w *Cnot3*$^{-/-}$ livers. Enriched GO terms of mRNAs upregulated (1) or downregulated (2) more than 2-fold in 1w *Cnot3*$^{-/-}$ livers compared to controls in microarray analysis were analyzed using DAVID. P-values, false discovery rates (FDRs) and gene lists included in GO terms are summarized. Please see also Supplementary figure 7A, C, D.

Click here to Download Table S1

Table S2. GO analysis of differently expressing mRNAs in 4w *Cnot3*$^{-/-}$ livers. Enriched GO terms of mRNAs upregulated (1) or downregulated (2) more than 2-fold in 4w *Cnot3*$^{-/-}$ livers in microarray analysis compared to controls were analyzed using DAVID. P-values, FDRs and gene lists included in GO terms are summarized. Please see also Figure 4A, C, D.

Click here to Download Table S2

Table S3. GO analysis of mRNAs which show both maturation-dependent downregulation in control livers and upregulation in 4w *Cnot3*$^{-/-}$ livers.
Enriched GO terms of mRNAs which show both more than 3-fold decrease from 1 to 4w in control livers and more than 2-fold upregulation in 4w *Cnot3*$^{-/-}$ livers were analyzed using DAVID. P-values, FDRs and gene lists included in GO terms are summarized. Please see also Supplementary figure 9.

Click here to Download Table S3

Development • Supplementary information

Table S4. Comparison of mRNA half-lives in control and *Cnot3*$^{-/-}$ fetal hepatocytes. Half-lives of mRNAs were calculated using the results of microarray analysis of differentiated fetal hepatocytes, which were treated with Act. D. The lists of mRNAs having more than 2-fold longer half-lives in *Cnot3*$^{-/-}$ fetal hepatocytes (Cnot3KO) than controls (Control) are shown together with the half-lives (T1/2) in control and *Cnot3*$^{-/-}$ fetal hepatocytes, ratios of half-lives (Cnot3KO/Control), gene symbols, Entrez Gene IDs, and gene titles. Please see also Supplementary figure 10E.

Click here to Download Table S4

Table S5. GO analysis of stabilized mRNAs in *Cnot3*$^{-/-}$ fetal hepatocytes. Enriched GO terms of mRNAs having more than 2-fold longer half-lives in *Cnot3*$^{-/-}$ fetal hepatocytes than controls (shown in Supplementary table 4) were analyzed using DAVID. P-values, FDRs and gene lists included in GO terms are summarized. Please see also Supplementary figure 10F.

Click here to Download Table S5

Table S6. GO analysis of mRNAs showing stabilization, transcriptional upregulation and downregulation in *Cnot3*$^{-/-}$ livers.

Enriched GO terms of mRNAs showing both upregulation and stabilization more than 1.5-fold (1), those showing both mRNA and pre-mRNA upregulation more than 1.5-fold (2), and those showing both mRNA and pre-mRNA downregulation more than 1.5-fold (3) in *Cnot3*$^{-/-}$ livers were analyzed using DAVID. P-values, FDRs and gene lists included in GO terms are summarized. Please see also Figure 7B, C and Supplementary figure 12.

Click here to Download Table S6

Table S7. Sequence lists of primers used in qPCR and polyA tail analyses, and siRNAs.

Click here to Download Table S7
